# Tissue-Specific Target Analysis of Disease-Associated MicroRNAs in Human Signaling Pathways

**DOI:** 10.1371/journal.pone.0011154

**Published:** 2010-06-30

**Authors:** Andreas Kowarsch, Carsten Marr, Daniel Schmidl, Andreas Ruepp, Fabian J. Theis

**Affiliations:** 1 Institute for Bioinformatics and Systems Biology, Helmholtz Zentrum München, Neuherberg, Germany; 2 Institute for Mathematical Sciences, Technische Universität München, Garching, Germany; John Innes Centre, United Kingdom

## Abstract

MicroRNAs are a large class of post-transcriptional regulators that bind to the 3′ untranslated region of messenger RNAs. They play a critical role in many cellular processes and have been linked to the control of signal transduction pathways. Recent studies indicate that microRNAs can function as tumor suppressors or even as oncogenes when aberrantly expressed. For more general insights of disease-associated microRNAs, we analyzed their impact on human signaling pathways from two perspectives. On a global scale, we found a core set of signaling pathways with enriched tissue-specific microRNA targets across diseases. The function of these pathways reflects the affinity of microRNAs to regulate cellular processes associated with apoptosis, proliferation or development. Comparing cancer and non-cancer related microRNAs, we found no significant differences between both groups. To unveil the interaction and regulation of microRNAs on signaling pathways locally, we analyzed the cellular location and process type of disease-associated microRNA targets and proteins. While disease-associated proteins are highly enriched in extracellular components of the pathway, microRNA targets are preferentially located in the nucleus. Moreover, targets of disease-associated microRNAs preferentially exhibit an inhibitory effect within the pathways in contrast to disease proteins. Our analysis provides systematic insights into the interaction of disease-associated microRNAs and signaling pathways and uncovers differences in cellular locations and process types of microRNA targets and disease-associated proteins.

## Introduction

MicroRNAs are endogenous, non-protein coding, approximately 22-nucleotide RNA molecules that have recently emerged as post-transcriptional regulators, known to influence diverse cellular processes ranging from stem cell differentiation to apoptosis [1]. They mostly target the 3′ untranslated region of a target mRNA, thereby destabilizing the transcript and inhibiting its translation [2], [3]. While there is evidence [4]–[6] that microRNA expression and maturation is induced by signaling pathways, microRNAs also emerge as regulators of signaling proteins. In zebrafish, miR-9 has been shown to regulate several components of the FGF signaling pathway, and thus controls neurogenesis in the midbrain-hindbrain domain during late embryonic development [7]. In another recent example in fruit fly [8], miR-8 has been identified to target both a transmembrane protein and a transcription factor of the WNT signaling pathway. Ricarte-Filho et al. [9] showed that the RET-pathway is mediated by let-7 which inhibits the activation of the RET/PTC-RAS-BRAF-ERK cascade exemplifying the direct influence of a single microRNA on a submodule of a signaling pathway. Given the generally large number of microRNA targets [10] it is natural to assume that many microRNAs regulate not only a single important pathway protein, but rather coordinate protein levels on a pathway-wide scale. Altered microRNA levels might then result in inaccurate target protein levels, consequently fallacious signal transduction, and potentially a disease phenotype.

From this perspective, it is intriguing to observe that medical sciences increasingly focus on the impact of microRNA-mediated regulatory control on diseases, especially in cancer: microRNAs are intensively used as diagnostic and prognostic disease markers [11], and even appear in first clinical trials [12]. Given the linkages between signaling pathways and microRNA regulation on the one hand, and microRNAs and disease phenotypes on the other, we aim to unveil the connection between phenotypes and pathways induced by microRNA mediated regulatory control.

In this work, we analyzed the tissue-specific regulatory patterns of disease-associated microRNAs in signaling pathways on different scales. Globally, we investigated the enrichment of disease-associated microRNAs on different pathways, and more locally, on the cellular location and process type of target proteins. We used manually annotated data from hundreds of patient studies to estimate the impact of disease-associated microRNAs on signaling pathways. We identified a core set of pathways, homogeneously enriched throughout nearly all diseases. Most of these pathways have been associated with cell growth, proliferation, and apoptosis. However, deregulation of signaling pathways can be induced by diverse factors. Point mutation of central signaling cascade proteins [13] have a severe impact on the information flow as well as any change in the expression pattern of *cis* or *trans* regulators. We thus compared the cellular localization and process type of signaling proteins that are microRNA targets with proteins that have been identified as disease-associated. In the following, we show that in contrast to disease proteins, microRNA targets are significantly enriched as inhibitors within the nucleus.

## Results

**Figure 1:**
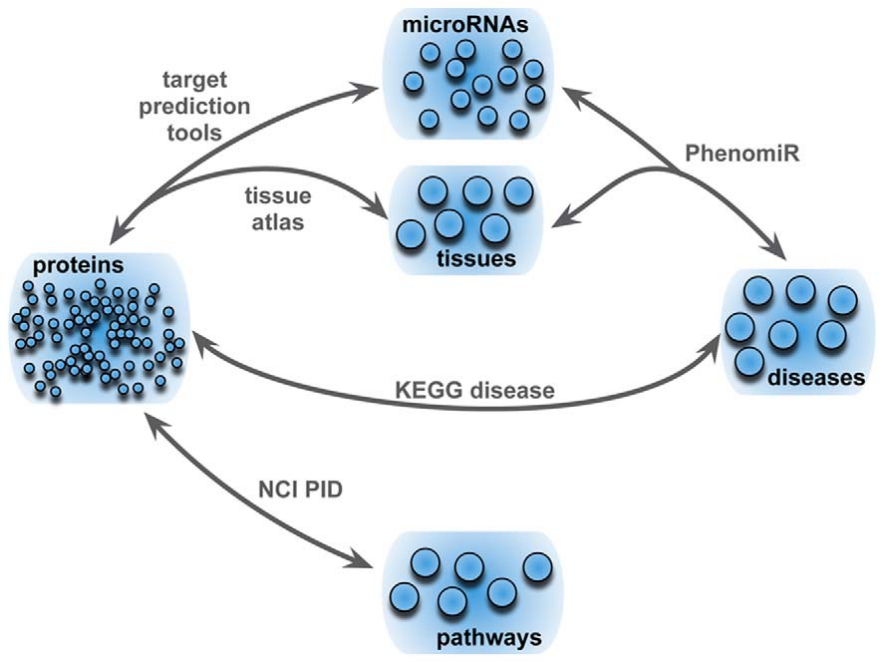
Illustration of the interactions between diseases, tissue, annotated disease-associated microRNAs, proteins, and human signaling pathways. The multipartite graphs consists of five sets of nodes and links between them, established by different data resources: 165 microRNAs from the PhenomiR database with annotated deregulation in 63 diseases, 4907 target transcripts, predicted by TargetScanS and filtered by the tissue atlas, 79 signaling pathways with constitutive proteins as given by the NCI PID database, and finally the subset of disease proteins as provided by the KEGG DISEASE database.

We captured the different entities of our investigation in a multipartite graph. The graph consists of five sets of nodes representing the entities microRNAs, proteins, tissue, diseases, and pathways and links between but not within the set of nodes. Links are given by a prediction tool and four databases. MicroRNAs (as provided by the mirBase database [14]) are linked to diseases and corresponding tissue via the PhenomiR database [15], a manually curated database containing disease-associated microRNAs in human disorders. MicroRNA target transcripts are determined by TargetScanS [10] a prediction tool that shows a high performance on different microRNA target data sets [16]. In addition, we used the tissue atlas provided by Su et al. [17] to filter potential microRNA targets for a specific disease and a given tissue. We unified the set of mRNA transcripts and corresponding proteins to a set of nodes denoted simply as proteins. This set is linked to signaling pathways via the National Cancer Institute Pathway Interaction Database (NCI PID) [18], containing 79 human pathways together with its constituting components. Finally, disease proteins are identified by their KEGG DISEASE annotation [19] (see Methods for a detailed description of the materials used). Figure 1 summarizes the entities and connections used. Notably, similar results were obtained with other microRNA prediction tools and a different set of disease genes, as provided by OMIM [20] (for a detailed discussion see Robustness analysis in File S1).

### MicroRNAs induce a core set of signaling pathways across diseases and tissues

**Figure 2:**
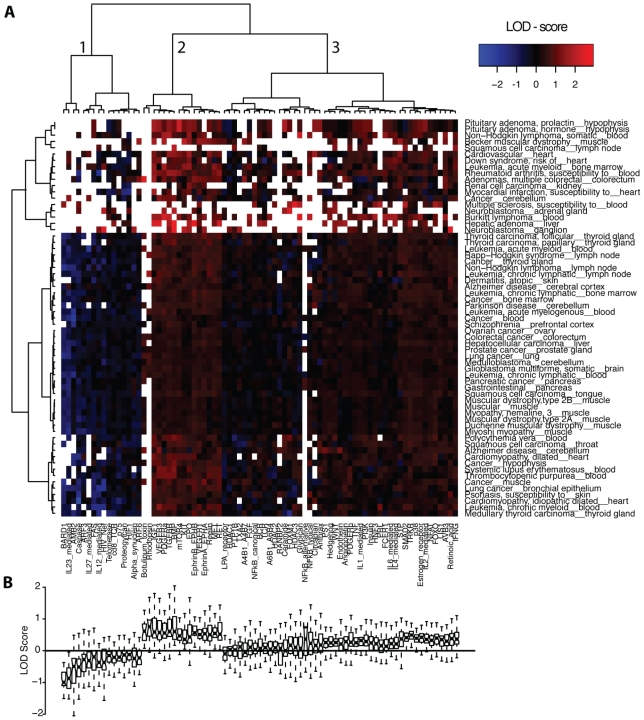
Impact of disease-associated microRNAs on signaling pathways. Enrichment for a particular disease and pathway was calculated by a LOD score. A positive score indicates an enrichment of microRNA targets for a disease-pathway interaction. Negative scores indicate depletion. **A**: Heatmap of microRNA target enrichment for a particular disease and pathway. Pathways and diseases are ordered by hierarchical clustering using Manhattan distance and ward clustering. **B**: Boxplot of disease-pathway associations ordered according to hierarchical clustering along the pathways. Red fields indicate an enrichments and blue a depletion. White fields indicate that no microRNA targets were found for this disease-pathway association.

We first analyzed the connection between diseases and signaling pathways, mediated by disease-associated microRNAs. In order to project the properties of the multipartite graph onto a disease-pathway correlation, we calculated the enrichment of disease-associated microRNA targets in a particular pathway. We used the tissue annotation in PhenomiR to filter for expressed microRNA targets, as given by the tissue atlas of Su et al. [17]. For a particular disease and a specific pathway, we computed the log odds ratio (LOD score) by dividing the relative number of associated microRNA targets in this pathway and tissue with the expected number, based on the relative number of associated microRNA targets in all signaling pathways given a specific tissue. Disease-pathway interactions with no targets (white fields in the heatmap Figure 2A) were excluded from further analyses (see Methods for a detailed description). We obtained a matrix of LOD scores, where each entry indicates the enrichment or depletion of tissue-specific targets of disease-associated microRNAs in the respective signaling pathway. We ordered this matrix according to a hierarchical clustering along the disease axis and pathway axis, respectively. Two features of the resulting heatmap are remarkable: First, dividing the hierarchical clustering of the signaling pathways into 3 major sub-clusters, we found one cluster (cluster 2; mean LOD = 0.55, variance = 0.008) showing a high enrichment throughout all diseases (see Figure 2A). We define this cluster as the core set of signaling pathways highly enriched with disease-associated microRNA targets. The remaining clusters show a high variance (cluster 3; mean LOD = 0.21, variance = 0.02) and a common depletion of microRNA targets (cluster 1; mean LOD = −0.36, variance = 0.07). Second, the 63 diseases split into two clusters with high and low microRNA-pathway associations. Within the larger of the two clusters, the enrichment of microRNA targets is extremely homogenous. Moreover we performed a multi-scale bootstrap resampling approach (relative sample sizes of bootstrap replication of 20%) [21] to test whether clusters 1–3 are robust against variation in the data. We can reject the hypothesis that the clusters do not exist with a significance level 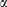
0.05 indicating that the clusters 1–3 may stably be observed by increasing the number of observations.

All signaling pathways located in the core set are given in Table 1. The functions of these pathways reflect the affinity of microRNAs to regulate cellular processes associated with apoptosis, proliferation or development, as we will outline with three examples. (i) The PDGFa pathway, for example, promotes cell migration, proliferation, and survival [22]–[25]. PDGF expression has been demonstrated in a number of different solid tumors, from glioblastomas to prostate carcinomas. Its biological function varies from autocrine stimulation of cell growth to subtler paracrine interactions involving adjacent stroma or vasculature [26]. (ii) It was recently reported that let-7 has an influence on the RET-pathway by effecting the cell growth and differentiation of papillary thyroid cancer [9]. Ricarte-Filho et al. [9] concluded that let-7 inhibited the activation of the RET/PTC-RAS-BRAF-ERK cascade exemplifying the direct influence of a single microRNA on a submodule of a signaling pathway. (iii) The Reelin pathway has been directly correlated with tumor aggressiveness [27]–[29]. Evangelisti et al. [30] linked this pathway for the first time to cancer by showing the inhibition of Reelin by miR-124a.

**Table 1:** Core set of signaling pathways with highly enriched microRNA targets.

Pathway	Median LOD	microRNA	Z-score 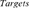	Z-score 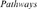
Rhodopsin	0.76	miR-154	8.69	6.58
Botulinum	0.61	miR-29b	8.58	8.10
TGFBR	0.61	miR-216a	12.20	7.10
BMP	0.60	miR-224	9.37	7.93
IGF1	0.59	miR-375	9.39	8.12
VEGFR3	0.57	miR-422a	8.29	7.89
EphrinB/EPHB	0.57	miR-422a	11.44	8.06
PDGFa	0.56	miR-383	7.20	7.59
MET	0.55	miR-422a	10.96	7.61
EphrinA/EPHA	0.53	miR-136	8.31	8.15
RET	0.52	miR-422a	9.04	7.24
VEGFR1	0.51	miR-422a	11.72	7.82
REELIN	0.51	miR-197	7.76	6.86
TRKR	0.49	miR-335	12.94	7.88
mTOR4	0.47	miR-375	7.23	7.44
EPO	0.43	miR-134	6.75	8.00

The Median LOD score is calculated over all diseases for a particular pathway. MicroRNA is the most enriched single microRNA within the corresponding pathway. Z-score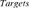 was calculated by comparing the median LOD score with the obtained score by a random sampling of microRNA targets. Z-score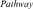 was calculated by comparing the median LOD score with the obtained score by a random sampling of pathway proteins.

The pathways with the highest negative enrichments, as depleted by disease-associated microRNA targets, are the IL-23 mediated pathway (playing a pivotal role in autoimmunity [31]) and BRAD1, which is associated with cell survival and cell death [32]. Although we found a core set of pathways across diseases, differences between disorders can arise due to different expression levels of the respective microRNAs. The PDGFa pathway for example shows high enrichments across diseases independent of the microRNA prediction tool (see Table S1). We found miR-144 to be highly enriched in the PDGFa pathway. Analyzing the expression profile, we found miR-144 down-regulated in cancer, but up-regulated in Parkinson disease and idiopathic Myelofibrosis. Predicted targets of miR-144 are SRF, a transcription factor activated by PDGFa, and FOS that is thought to have an important role in signal transduction, cell proliferation and differentiation [33]–[35]. This finding shows that although different diseases are associated with the same signaling pathway, differences in the effects of the stimulated pathways can be induced by complementary expression profiles of microRNAs.

As the PhenomiR data set is dominated by cancer-related diseases (60%), we divided the set of diseases into a subset of cancer and non-cancer related microRNAs to study differences between both groups. We found 14 out of 16 pathways of the global core set also in the cancer-specific core set (see Table S2). The core set for the non-cancer related pathways contains 12 pathways that were also found by the global data set, but we also identify also two non-cancer specific pathway enrichments (see Table S3) such as the KIT pathway and the NF
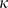
B pathway, that is involved in the expression of genes associated with development, cell death, and immune response [36]–[39].

### Robustness analysis of the core set of signaling pathways

In order to ensure that our results are not artifacts of the chosen prediction tool, we analyzed the data with four other prediction tools: PicTar [40], Miranda [41], TargetSpy [42], and RNA22 [43]. Different features like conservation of the seed region or binding energies are taken into account to predict microRNA-transcript interactions in each tool. Based on these differences the overlap between the target sets from different tools is generally rather low [44]. We define for each tool the core set of signaling pathways, which are highly enriched by microRNA targets and compare these list with our core set listed in Table 1. The result shows that the signaling pathways in our core set are mostly consistent with different prediction tools (see Table S1). We found 8 out of 16 pathways within the core set of at least 3 different prediction tools.

In order to test the significance of these pathways, we performed a randomization approach, by comparing the median LOD score of these pathways with the median scores obtained by two random samplings. We first sampled 10.000 times pathway proteins keeping the pathway size constant, second, we generated 10.000 times a random microRNA predictor by sampling for each microRNA the corresponding targets. Finally, we calculated a z-score to estimate the significance of each pathway within the core set. We obtained high z-scores for the pathways within the core set independent of the sampling approach (see Table 1). The mean z-score for all pathways is 12.51 (Z-score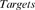) and 7.65 (Z-score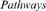), respectively.

The enrichment of microRNA targets is summarized in the boxplot in Figure 2B, where the distribution of LOD scores for each pathway is shown. The median LOD scores and their variance for the set of signaling pathways are significantly negatively correlated (Pearson correlation coefficient C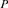 = 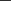
0.37, 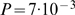, see Figure S1). In contrast to depleted pathways, highly enriched pathways are homogeneously targeted by microRNAs across diseases. This indicates that disease-associated microRNAs in human disorders target a core set of signaling pathways irrespective of the specific disease and tissue.

We ensure that the LOD scores are not trivially biased by the pathway size (C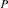 = −0.032, 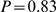) and show the respective plot in Figure S2. We noticed that the pathway enrichment is significantly negatively correlated with the number of microRNAs with targets in this pathway (C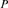 = −0.31, 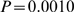), with up to 159 targeting microRNAs in the SMAD2 pathway.

### Interaction of disease-associated proteins and microRNA targets

Much effort has been invested in understanding the mechanisms underlying the complex network of factors contributing to human diseases. Databases like OMIM [20], KEGG DISEASE [19], or HGMD [45] link dysfunctional proteins and genetic mutations to human disorders. In order to focus on already confirmed gene-disease interactions, we used the KEGG DISEASE database to study similarities and differences to microRNA targets in signaling pathways. In the following, we analyzed 23 diseases that are both annotated in KEGG DISEASE and PhenomiR (see Methods). In this subset, we analyzed 365 KEGG DISEASE proteins located in the NCI PID signaling pathways and identified 123 (33.7%) proteins as microRNA targets. The current estimation for the amount of microRNA targets in the human genome lies between 30–35% [10], [46]. This implies that there is no higher rate of microRNA targets in the set of disease proteins than expected. In order to study the interplay of disease proteins and microRNA targets, we compared their mapping to NCI PID pathways (see Figure 1). We found that typically, disease-affected proteins are widely distributed over pathways for a particular disease. Focusing on pathways showing a high fraction of disease-associated proteins, we found no correlation of microRNA target enrichment and the fraction of disease-affected signaling proteins (see Figure S3). These findings imply that disease-affected proteins and disease-associated microRNA targets do not prefer a common set of signaling pathways. To elucidate those differences, we changed the scale of our investigation and compare the localization and process type of disease-associated microRNA targets and disease proteins.

### MicroRNA targets are preferentially located in the nucleus in contrast to disease proteins

**Figure 3:**
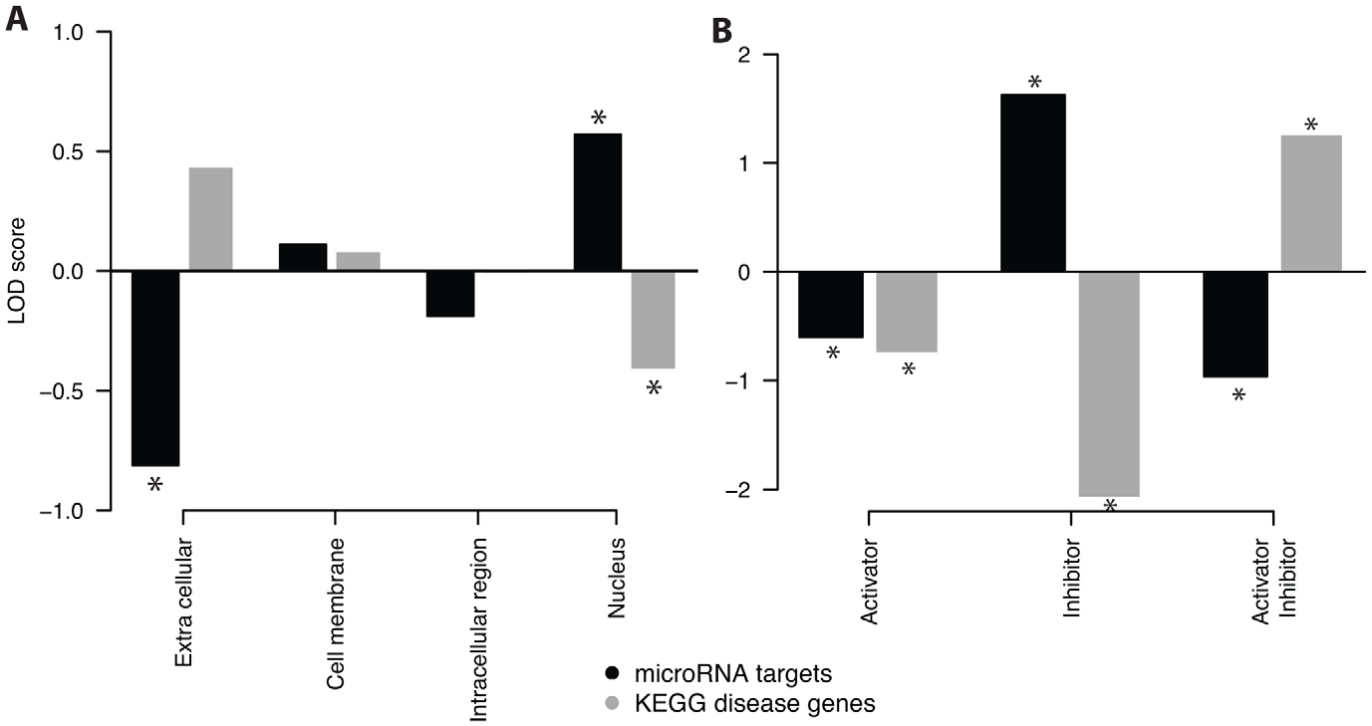
Analysis of cellular location and process type distribution for microRNA targets and disease proteins. **A**: Signaling proteins are divided into four different cellular location groups (extracellular region, cell membrane, intracellular region, and nucleus) based on their NCI PID annotation. We calculated the enrichment of microRNA targets and disease proteins by a LOD score. We found an opposing patterns of cellular localization for disease-associated proteins and microRNA targets. **B**: Process type information obtained by the NCI PID database was used to divide signaling proteins into three different groups, activators, inhibitors, and ambivalent proteins (annotated as both activators and inhibitors). The result indicates again complementary patterns for microRNA targets and human disease proteins. * indicates significant enrichment obtained by Fisher's exact test (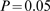).

To question whether microRNA targets and KEGG DISEASE proteins differ with respect to their cellular location and process type annotation, we divided the set of signaling proteins according to their NCI PID annotation into four groups: extracellular region, cell membrane, intracellular region, and nucleus. We then estimated the fraction of microRNA targets as well as disease proteins for each group and calculated the LOD enrichment scores (see Methods for a detailed description). Surprisingly, we found opposing patterns of cellular localization for disease-associated proteins and microRNA targets (see Figure 3A). Deregulated microRNAs preferentially target nuclear proteins (LOD = 0.57, 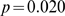), while disease-associated proteins in the nucleus are underrepresented (LOD = −0.41, 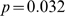). Therefore, microRNA targets are almost twice more frequently located in the nucleus as compared to disease proteins. Furthermore, proteins located in extracellular region are only weakly controlled (LOD = −0.81, 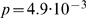) by microRNAs. Disease associated proteins showing again a complementary result compared to microRNA targets (LOD = 0.44, 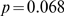), being more than twice more frequently located in the extracellular region. Proteins located in the cell membrane or intracellular region show no significant differences and enrichments for microRNAs or disease-associations. Comparing these results with the subset of cancer-related microRNAs we obtained the similar finding of a preferred target location in the nucleus. This result shows that preferred location is not based on a disease-specific set but a common pattern, valid for cancer as well as non-cancer related microRNAs (see Figure S4). We repeated the location analysis with different prediction tools and obtained similar results for microRNA targets (see Figure S5). Analyzing microRNA targets located in the nucleus by Gene Ontology, we found 50% of those genes involved in transcriptional regulation. In addition, we used the OMIM database to select disease-associated genes and found again a opposite pattern of cellular localization for OMIM and microRNA targets (see Figure S6).

### In contrast to disease proteins, microRNA targets frequently exhibit an inhibitory effect

We sorted the set of signaling proteins into three different groups according to their process type annotation: activating proteins, inhibiting proteins and proteins that can act as either activators or inhibitors, further on denoted as ambivalent. We then counted the number of microRNA targets as well as disease proteins for each group in our signaling pathways and calculated the LOD score. The result shows again a complementary pattern: As shown in Figure 3B, targets of disease-associated microRNAs are preferentially inhibitors (LOD = 1.62, 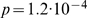), whereas only 6 disease-associated proteins (LOD = −2.08, 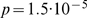) show a inhibitory effect. MicroRNA targets are enriched almost 14 times more in inhibiting proteins compared to disease proteins showing a complementary focus. Ambivalent proteins show a strong under-representation for microRNA targets (LOD = −0.96, 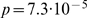), whereas disease-affected proteins are significantly enriched (LOD = 1.26, 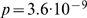). For activators, we found a significant under-representation for both disease proteins (LOD = −0.75, 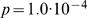), and microRNA targets (LOD = −0.60, 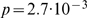), respectively. Again, we found the same result for cancer and non-cancer related microRNA targets indicating a common pattern. Notably, the enrichment of process types of disease proteins remains for the OMIM data set (see Figure S7).

## Discussion

In order to study the role of disease-associated microRNAs in pathways, we applied a thorough statistical analysis to a multipartite graph consisting of microRNAs, proteins, diseases, tissue and signaling pathways. We investigated enrichment of disease-associated microRNAs globally on different pathways by considering of tissue-specific transcript expression, and more locally, on the cellular location and process type of target proteins.

We found that the amount of regulatory control mediated by disease-associated microRNAs differs from pathway to pathway. In [47], the authors showed that the targets of a specific microRNA cluster are significantly enriched in multiple pathways. For the majority of diseases, a homogeneous enrichment profile of microRNA targets throughout all pathways emerged. From our analysis of the constituting multipartite graph, we found that pathways are heterogeneously targeted by microRNAs. However, the core set of pathways under strong microRNA control appear to be homogeneously enriched throughout the majority of diseases, since many diseases are linked to a large number of microRNAs. So far, almost two third of the currently known microRNAs are linked via large-scale expression analysis to a phenotype. It is obvious that beside the phenotype responsible microRNAs, many microRNAs are detected as deregulated in human diseases but are not functionally linked to the phenotype.

What could be the biological function of a core set of globally enriched pathways? We showed that these pathways are targets of numerous deregulated microRNAs. One possible hypothesis is that these pathways could serve as disease sensors, transferring the information of erroneous cellular functions via deregulated microRNAs to important output proteins, like cell cycle checkpoints. From this perspective, it is intriguing that most top enriched pathways are associated with apoptotic, proliferation or developmental processes [48]. Entries in the PhenomiR database obtained by patient studies are more than 60% cancer-related diseases. Alterations in the expression or function of genes controlling cell growth and differentiation are considered to be the major cause of cancer. Notably, degenerative disorders like Alzheimer or Parkinson disease show a similar pathway profile compared to cancer-related phenotypes, although often with different direction of microRNA expression.

Presumably, the impact on signaling pathways for disease-associated proteins and microRNA targets differs. However, there might be an interaction between the disease-associated microRNAs and proteins to mediate deregulation of signaling pathways. It would be interesting to evaluate whether a given disease emerges due to protein deregulation caused by mutations with a successive deregulation of microRNAs, or due to deregulated microRNA levels, leading to pathogenic protein levels in turn. For a subset of microRNAs, located in the intron of a host gene, an examination of a common phenotypic effects is possible. Recently, we showed that intronic microRNAs support the regulatory effect of their host genes [49]. Here, we find one disease-associated microRNA-target pair with a common phenotype: both the host gene PTK2 and its intronic microRNA miR-151 are annotated with lung cancer in KEGG DISEASE and PhenomiR, respectively. In this case, the impact on the associated signaling pathways via correlated mir-151 and PTK2 deregulation is probably controlled by a single promoter. To unveil interactions between microRNAs and pathway proteins on a systems level, a much more precise knowledge of microRNA transcriptional regulation is needed.

We analyzed the subcellular location and process type behavior of disease-associated proteins and microRNA targets. Our result on the preferred cellular locations of microRNA targets shows an enrichment of proteins in the nucleus. This finding is in line with a study by Cui et al. [50], who obtained a similar result for the localization of microRNA targets on a much smaller set of signaling networks and microRNAs in mammalian hippocampal CA1 neurons. In addition, we found that disease-associated proteins often constitute the initial players of signaling networks and thus show an opposite pattern to microRNA targets. The deregulation of a single proteins at the cell surface receptor can have a severe impact on the whole signaling information flow stimulated by the receptor. For example, for growth factor receptors, the activation under normal conditions promotes cellular survival, whereas over-expression promotes tumor cell growth [51]. Therefore, cell surface receptors are well suited as drug targets, as diminishing the signal through these receptors has the potential to normalize cellular behavior. The deregulation of a single protein in the intracellular region or the nucleus might influence only a subpart of the signaling network.

A large fraction (50%) of microRNA targets located in the nucleus are involved in transcriptional regulation. It was shown that transcription factors like MYC, JUN, or FOS, have a short mRNA lifetime based on their RNA stability [52], [53]. Within these studies the importance of the 3′ untranslated region for the mRNA stability was mentioned. Thus, microRNAs presumably tune RNA stability in a tissue or stage dependent manner. Deregulated microRNAs changing the stability of transcription factors of a signaling pathway may then lead to malfunction of different cellular processes [54]. Motivated by the affinity of microRNAs to regulate with associated pathways apoptosis, proliferation or development [1], we suppose that the regulation of stability extends to proteins with short half-lives that are required only for limited time in, e.g., cell cycle, growth, or differentiation.

In a recent study, Legewie et al. [55] introduced a set of signal inhibitors with a short mRNA and protein lifetime that are transcriptionally induced upon stimulation. These rapid feedback inhibitors (RFIs) are thought to tune the signal transduction cascades, allow for swift feedback regulation and establish short latency phases after signaling induction. As we found an enrichment of inhibitory proteins targeted by microRNAs, the question arises, if RFI proteins are potential microRNA targets. Using the TargetScanS prediction tool we were able to confirm 18 out of 19 (95%) RFIs as microRNA targets (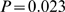). We thus assume that the short mRNA lifetime of RFIs can be attributed to the degradation activity promoted by microRNA binding. Inhibiting proteins are preferentially located in the nucleus (see Table S4), whereas activating or ambivalent proteins are randomly distributed in the cellular regions. Interestingly, disease proteins showed a frequent association with ambivalent process type. We assume that for ambivalent proteins, deregulation of the expression levels imparts a more severe effect on signaling cascades as compared to activators or inhibitors alone.

The usage of hypergraphs for a proper representation of interconnected entities in systems biology has been acknowledged recently [56]. Here, we applied a thorough statistical analysis not only to bipartite but to a multipartite graph consisting of microRNAs, proteins, diseases, and signaling pathways in a tissue-specific manner and uncovered the impact of disease-associated microRNAs on human signaling pathways.

## Materials and Methods

In this section, we give a detailed overview about the resources and methods, which were used to interconnect the different entities shown in Figure 1.

### Human signaling pathway data

Human signaling pathway data was obtained from the National Cancer Institute Pathway Interaction Database (NCI PID) [18], which is a manually curated collection of biomolecular interactions and key cellular processes assembled into signaling pathways. NCI PID holds 128 pathways including 47 sub-networks. We combined all subnetworks with their parent networks to the set of signaling pathways. In addition, we kept all pathways that have more than one predicted microRNA target gene, leading to a final data set of 79 human signaling pathways containing 1573 unique human proteins. The database also provides information on subcellular location terms from the Gene Ontology Consortium. We used this information to divide all subcellular locations into four different groups: extracellular region, cell membrane, intracellular region and nucleus. Finally, location information for 1083 proteins containing 135 extracellular region, 344 cell membrane, 373 intracellular region and 231 proteins located in the nucleus were obtained. In addition, we extracted process type information for each biological process, which can be input, output, positive or negative regulator. In total, there are 1120 interactions of which 765 are activating, 74 inhibiting and 281 proteins acting as activators as well as inhibitors.

### Disease-associated microRNAs

Human disease-associated microRNAs were obtained from the PhenomiR database [15]. PhenomiR is a manually curated collection of microRNA-disease associations, containing a total of 11 029 microRNA expression-phenotype relations collected from 542 different experiments. We used patient study data only and obtained 486 disease-associated microRNAs in 83 different diseases including up to 5 subtypes per disorder. For each disease, we take only those microRNA into account, that have at least one target in the specific tissue annotated by PhenomiR and obtained finally 165 different microRNAs in 63 diseases-tissue combinations.

### MicroRNA target prediction

Hausser et al. [16] analyzed different features of microRNA targets and showed within their work that TargetScanS has a good performance on different data sets. We used TargetScanS as the main prediction tool but to handle the issue of the unknown reliability of microRNA prediction tools we used several other prediction tools like PicTar, intersection of PicTar and TargetScanS, Miranda, RNA22, and TargetSpy to confirm our results. We used for each method default parameter settings.

### MicroRNA targets filtered by tissue expression

As microRNA expression is tissue-specific annotated in PhenomiR, we used the tissue atlas provided by Su et al. [17] to filter potential microRNA targets in a specific tissue. The data was downloaded from the NCBI Gene Expression Omnibus (GEO), and the processed data was used. We mapped the predicted microRNA target transcripts on the tissue atlas and considered a transcript as expressed in a specific tissue, if either one replicate has a present call or both show at least a marginal call, similar to the work of McClintick et al. [57].

### Human disease data

Human disease proteins were taken from the KEGG DISEASE database [19]. It associates 5 neurodegenerative disorders, 5 infectious and metabolic disorders and 13 different cancer diseases. Finally, we obtained 909 proteins from 23 different diseases, which are also found in the PhenomiR database. For results obtained by the NCBI OMIM database see Figure S6 and Figure S7.

### Pathway profile

Pathway profiles were calculated for all diseases annotated in PhenomiR passing the tissue filter. For each disease-pathway interaction we estimated the enrichment of microRNA targets of disease 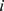 in pathway 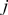 defined by a log odds ratio (LOD score):
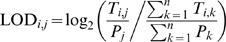
where 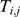 is the number of microRNA targets for all disease-associated microRNAs in disease 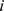 and pathway 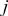; 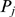 is the number of proteins in pathway 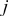; 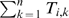 is the number of microRNA targets for all disease-associated microRNAs in disease 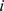 over all pathways; 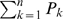: is the number of proteins over all pathways. We use these LOD scores to build up a heatmap using Manhattan distance function and ward clustering. A positive value indicates an enrichments and a negative a depletion. Whenever we identified no target for a particular disease-pathway interaction 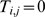 and therefore the resulting LOD score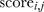 is 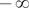. As commonly done, we excluded all cases with 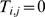 for calculating the mean and quantiles for each pathway. In addition, these cases were also excluded from the clustering taking the reduced dimensions into account.

### Cellular location analysis

We used the subcellular location annotation of the NPI PID database to estimate the microRNA target enrichment. The enrichment was calculated by the logarithm of base 2 of the odds ratio (LOD score) and its significants was obtained by Fisher's exact test.

### Process type analysis

In addition to the subcellular location, the NPI database provides information about specific process types of proteins in signaling processes. We used this information to analyze the interaction between inhibiting as well as activating proteins in signaling processes. Within this analysis we calculated the enrichment of microRNA targets as well as KEGG DISEASE proteins for different process types. The enrichment was calculated by the logarithm of base 2 of the odds ratio (LOD score) and its significants was obtained by Fisher's exact test.

## Supporting Information

File S1 Robustness analysis.
(13.55 MB PDF)

Figure S1 Anticorrelation of median LOD score against variance for signaling pathways. We obtained a significant negative correlation (Pearson correlation coefficient Cp = −0.37, P = 0.007). The result implies that deregulated microRNAs in human diseases target the same set of signaling pathways irrespective of the specific disorder. The results of the linear regressions is shown by the black line.
(0.12 MB TIF)

Figure S2 Pathway size against pathways ordered by median LOD score. We found no correlation (Cp = −0.032, P = 0.83) between pathway size against pathways ordered by median LOD score. The results of the linear regressions is shown by the black line.
(0.15 MB TIF)

Figure S3 Correlation between KEGG DISEASE proteins and microRNAs in signaling pathways, using 24 diseases both annotated in PhenomiR and KEGG DISEASE. Median LOD score of signaling pathways against the fraction of disease-associated pathway proteins (Cp = 0.14, P = 0.21). We consider all pathways showing a fraction of disease-associated proteins ≥0.2. We observe no significant correlation between increasing LOD scores and the fraction of disease proteins even if we exclude the outlier (marked in red) (Cp = 0.18, P = 0.127). The results of the linear regressions is shown by the black line.
(0.15 MB TIF)

Figure S4 Comparison between different disease sets. Observed LOD scores for cellular location of all disease-associated microRNA targets and two subsets of diseases (Cancer, Non Cancer) using TargetScanS. For cancer and non cancer, we observed similar scores compared to scores obtained by using all diseases showing that the location pattern is rather a common result and not depended on the subsets of cancer and non-cancer related microRNAs.
(0.14 MB TIF)

Figure S5 Comparison between different microRNA prediction tools. Observed LOD scores for cellular location of several microRNA prediction methods (Intersection of PicTar and TargetScanS, TargetScanS, PicTar, Miranda, TargetSpy, and RNA22) and KEGG DISEASE proteins. Different features like conservation of the seed region (e.g., TargetScanS) as well as binding energies (e.g., Miranda) are taken into account to predict microRNA-transcript interactions. Based on differences in these prediction methods the overlap between the targets from different tools is low (Sethupathy, 2006). In this work, it was also shown that Miranda has similar high sensitivity compared to the top method like TargetScanS, but exhibit a substantial increase in the number of total predictions. This could be one explanation why Miranda shows a different result for microRNA targets in extracellular and intracellular regions compared to the remaining prediction tools, which show very similar results. The findings indicate robustness of our results, independent on the prediction tools. In addition, this findings support our result of complementary behavior of KEGG DISEASE proteins and microRNA targets.
(0.19 MB TIF)

Figure S6 Comparison between different disease gene sets. Observed LOD scores for cellular location of microRNA targets and two sets of disease-associated genes (KEGG DISEASE and OMIM). For OMIM, we observed similar scores compared to KEGG DISEASE proteins that confirms our finding and shows robustness of our results. In addition, this finding supports our result of complementary behavior of disease-associated genes (KEGG DISEASE and OMIM) and microRNA targets.
(0.15 MB TIF)

Figure S7 Comparison between different disease gene sets. Observed LOD scores for process type behavior of microRNA targets and two sets of disease-associated genes (KEGG DISEASE and OMIM). For OMIM, we observed similar scores compared to KEGG DISEASE proteins that confirms our finding. In addition, this finding supports our result of complementary behavior of disease-associated genes (KEGG DISEASE and OMIM) and microRNA targets.
(0.15 MB TIF)

Table S1 Core set of signaling pathways. Prediction tools show the fraction of different tools having the corresponding pathway within the top cluster.
(0.02 MB XLS)

Table S2 Core set of signaling pathways obtained by the cancer related microRNAs. Prediction tools show the fraction of different tools having the corresponding pathway within the top cluster.
(0.02 MB XLS)

Table S3 Core set of signaling pathways obtained by the non-cancer related microRNAs. Prediction tool shows the fraction of different tools having the corresponding pathway within the top cluster.
(0.02 MB XLS)

Table S4 Correlation between cellular location and process type. Number of signaling proteins within the four different cellular locations and three different process types.
(0.03 MB XLS)
