# Supplementary material for: Tissue-Specific Target Analysis of Disease-Associated MicroRNAs in Human Signaling Pathways
Source: PLoS One. 2010 Jun 30;5(6):e11154. doi: 10.1371/journal.pone.0011154 (PMC2894853; doi:10.1371/journal.pone.0011154)
Supplement: File S1 — Robustness analysis. (13.55 MB PDF) [file pone.0011154.s001.pdf]

Supplementary Information

Tissue-specific target analysis of disease-associated microRNAs in human signaling pathways

Andreas Kowarsch[1], Carsten Marr[1], Daniel Schmidl[1,2], Andreas Ruepp[1], Fabian J. Theis[1,2]

[1] Institute for Bioinformatics and Systems Biology, Helmholtz Zentrum München, Neuherberg, Germany

[2] Institute for Mathematical Sciences, Technische Universität München, Garching, Germany

Table of contents

Robustness analysis **2**

Supplementary Figure 1: Impact of disease-associated microRNAs on signaling pathways obtained by PicTar. 3

Supplementary Figure 2: Impact of disease-associated microRNAs on signaling pathways obtained by the intersection of PicTar and TargetScanS. 4

Supplementary Figure 3: Impact of disease-associated microRNAs on signaling pathways obtained by Miranda. 5

Supplementary Figure 4: Impact of disease-associated microRNAs on signaling pathways obtained by TargetSpy. 6

Supplementary Figure 5: Impact of disease-associated microRNAs on signaling pathways obtained by RNA22. 7

Supplementary Table 1: Core set of signaling pathways. 8

Supplementary Table 2: Core set of signaling pathways obtained by the cancer related microRNAs. 8

Supplementary Table3: Core set of signaling pathways obtained by the non-cancer related microRNAs. 9

Cellular location analysis 10

Supplementary Figure 6: Comparison between different microRNA prediction tools . . 10

Supplementary Figure 7: Comparison between different disease sets 11

Supplementary Figure 8: Comparison between different disease gene sets 12

Process type analysis 13

Supplementary Figure 9: Process type analysis using different microRNA prediction tools 13

Supplementary Figure 10: Comparison between different disease sets 14

Supplementary Figure 11: Comparison between different disease gene sets 15

Robustness analysis

The accuracy of microRNA prediction tools has already been demonstrated by experimental validation. Hausser et al. [1] analyzed different features of miRNA targets and showed within their work that TargetScanS has a good performance on different data sets. Moreover, there are indications that the combination of different methods yields an increase in sensitivity [2]. Different techniques like conservation of the seed region as well as binding energies between microRNA and the 3'-UTR are taken into account to predict microRNA-gene interactions. Based on differences in these prediction methods the overlap between the targets from different tools is low [3]. In order to test the potential error introduced by the microRNA target gene data set and to validate our findings, we performed a robustness analysis using five different microRNA target prediction methods: PicTar, TargetScanS, Miranda TargetSpy, and RNA22.

Supplementary Figure 1 to 5 show the heatmaps and corresponding boxplots for each prediction tool using the PhenomiR data set. Enrichment for a particular disease and pathway was calculated by a LOD score. A positive score indicates an enrichment of microRNA targets for a disease-pathway interaction. Negative scores indicate depletion. Pathways and diseases are ordered by hierarchical clustering using Manhattan distance and ward clustering. Red fields indicate an enrichments and blue a depletion. White fields indicate that no microRNA targets were found for this disease-pathway association.

Supplementary Figure 1: Impact of disease-associated microRNAs on signaling pathways obtained by PicTar.

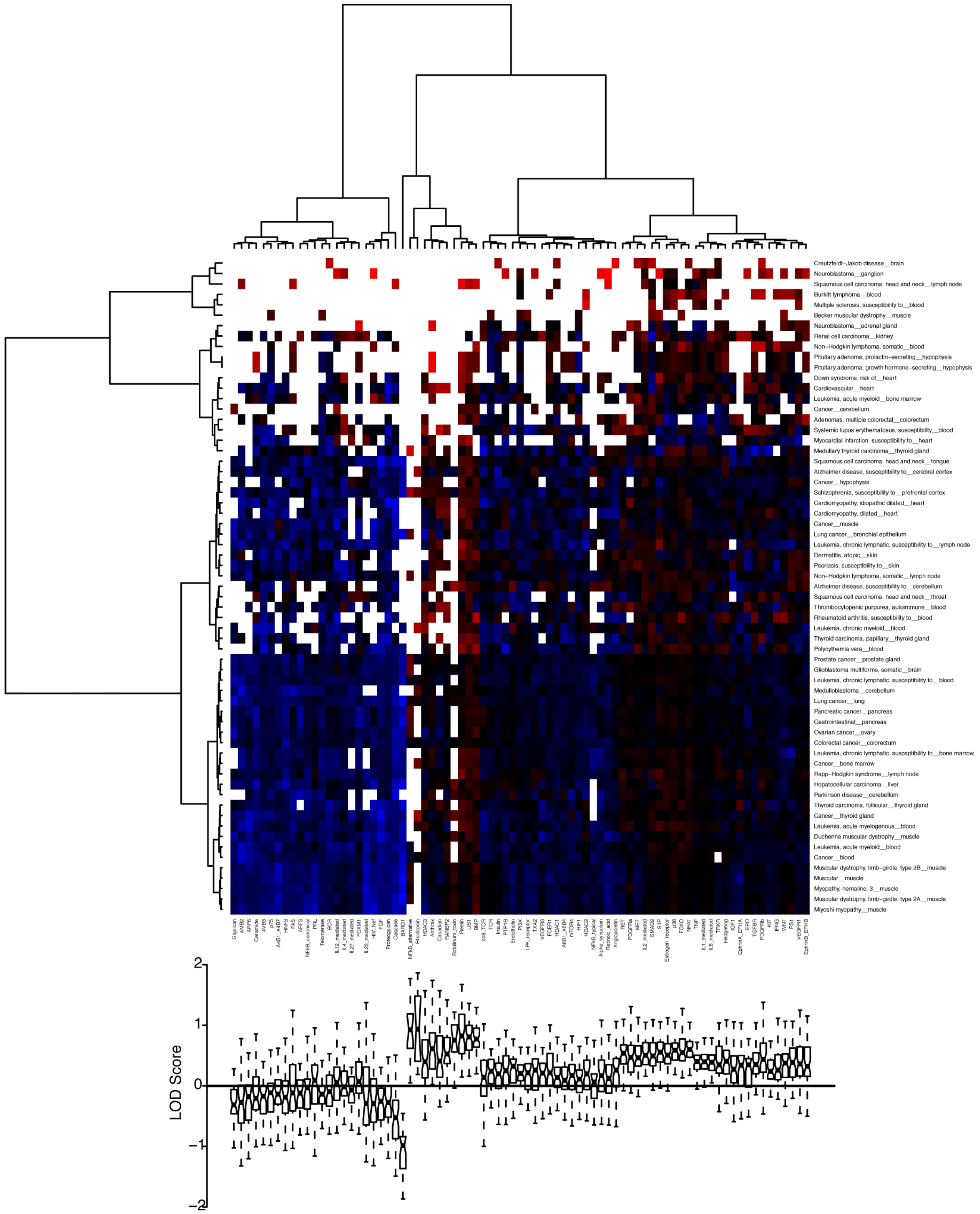

Supplementary Figure 2: Impact of disease-associated microRNAs on signaling pathways obtained by the intersection of PicTar and TargetScanS.

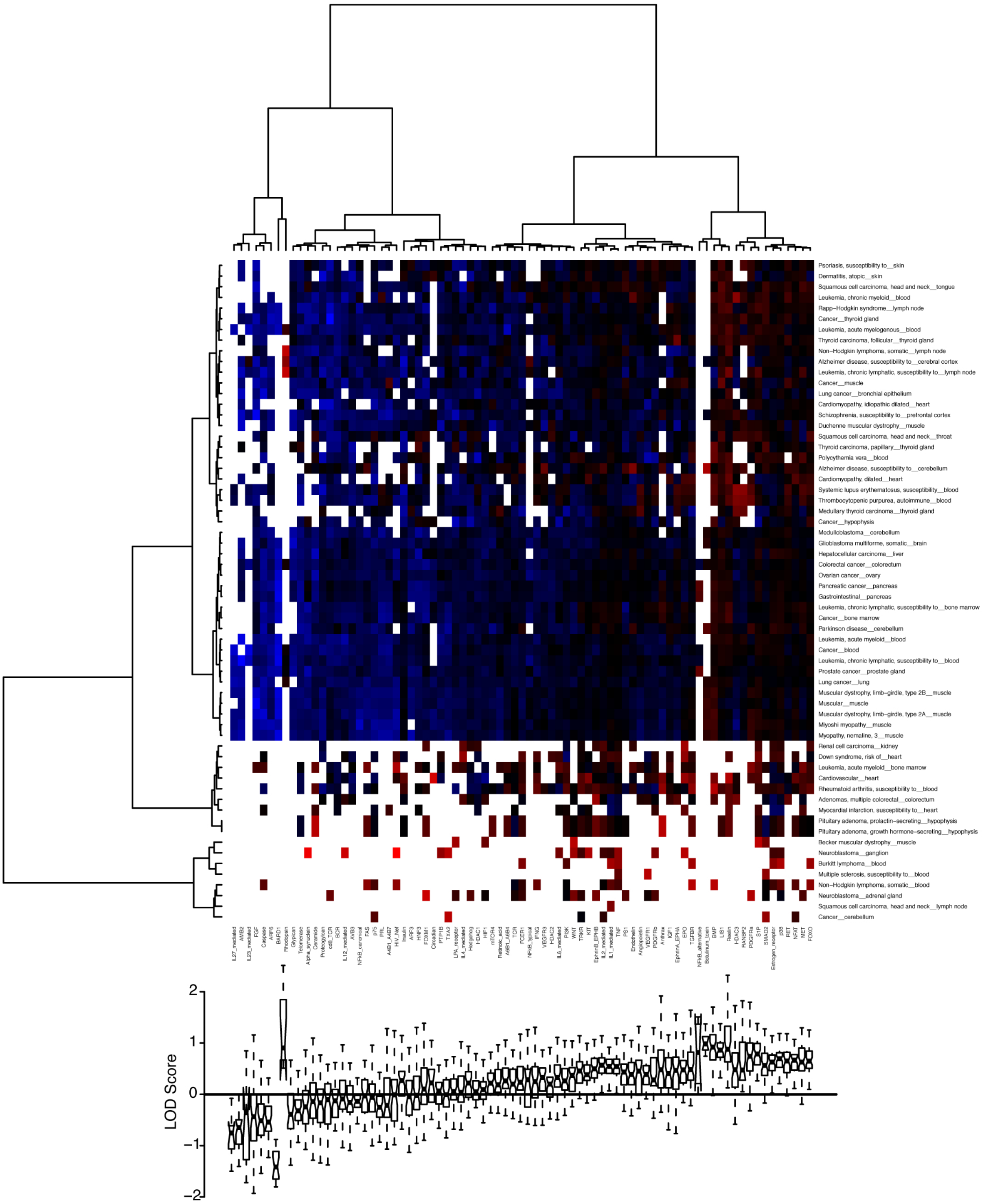

Supplementary Figure 3: Impact of disease-associated microRNAs on signaling pathways obtained by Miranda.

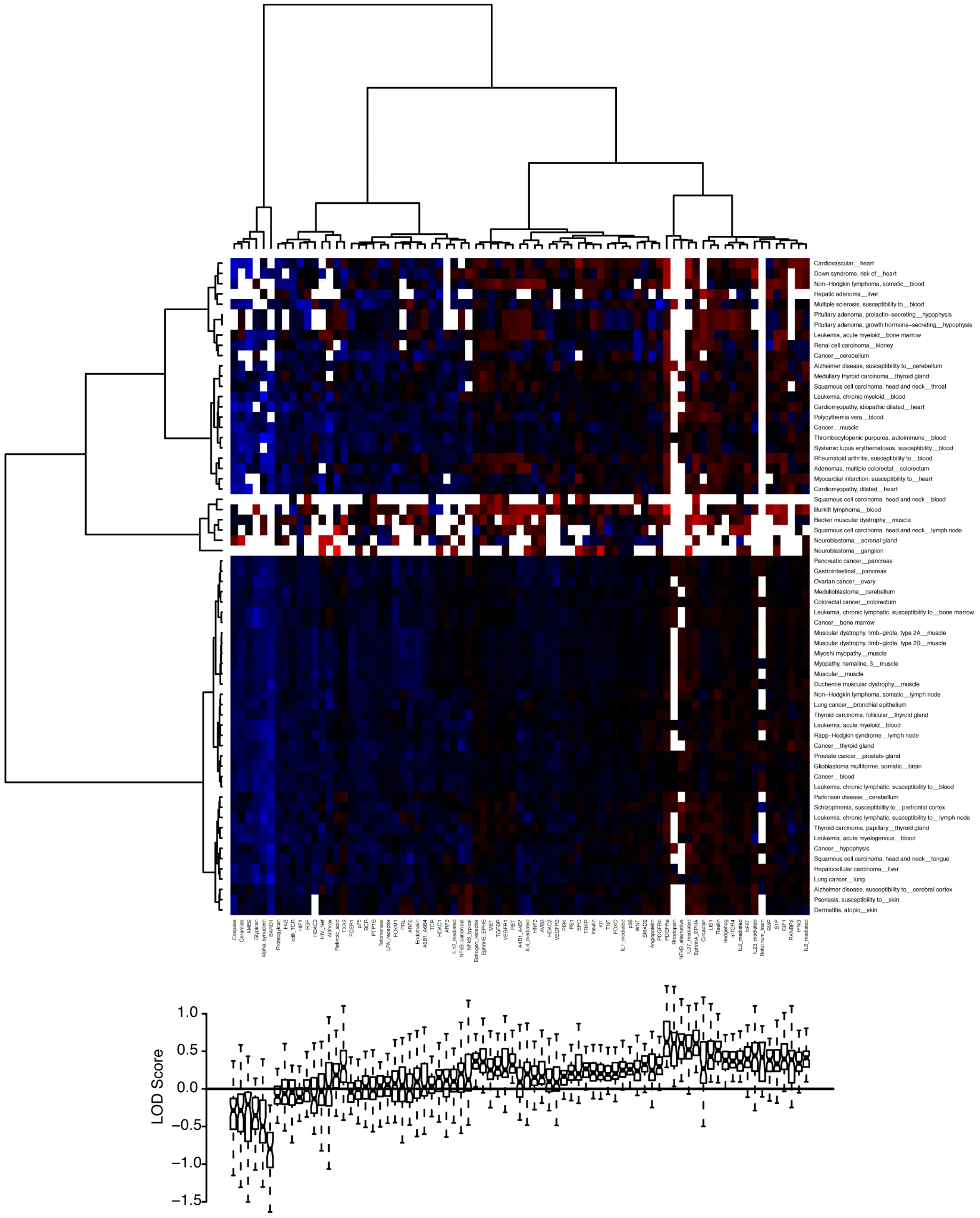

Supplementary Figure 4: Impact of disease-associated microRNAs on signaling pathways obtained by TargetSpy.

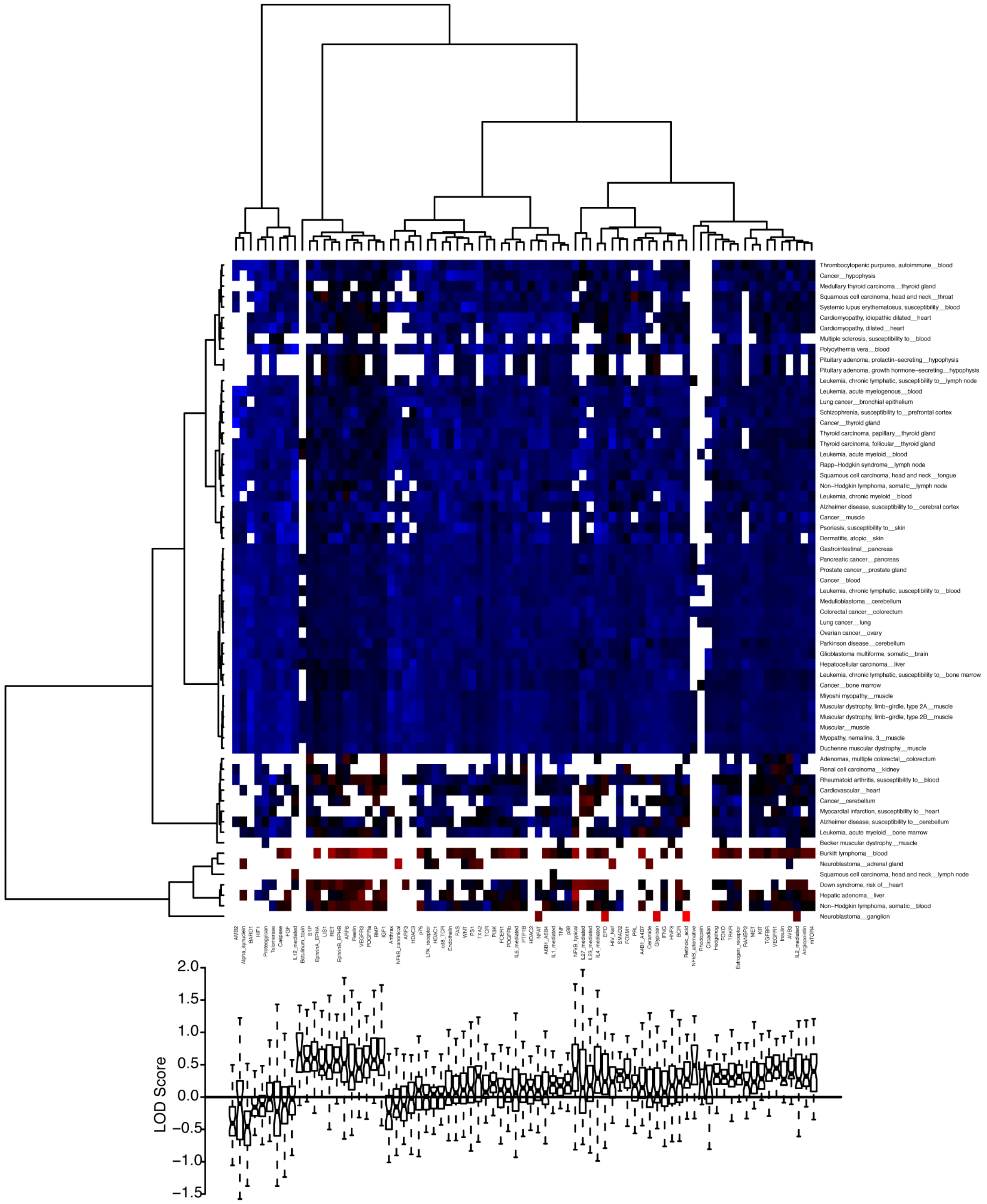

Supplementary Figure 5: Impact of disease-associated microRNAs on signaling pathways obtained by RNA22.

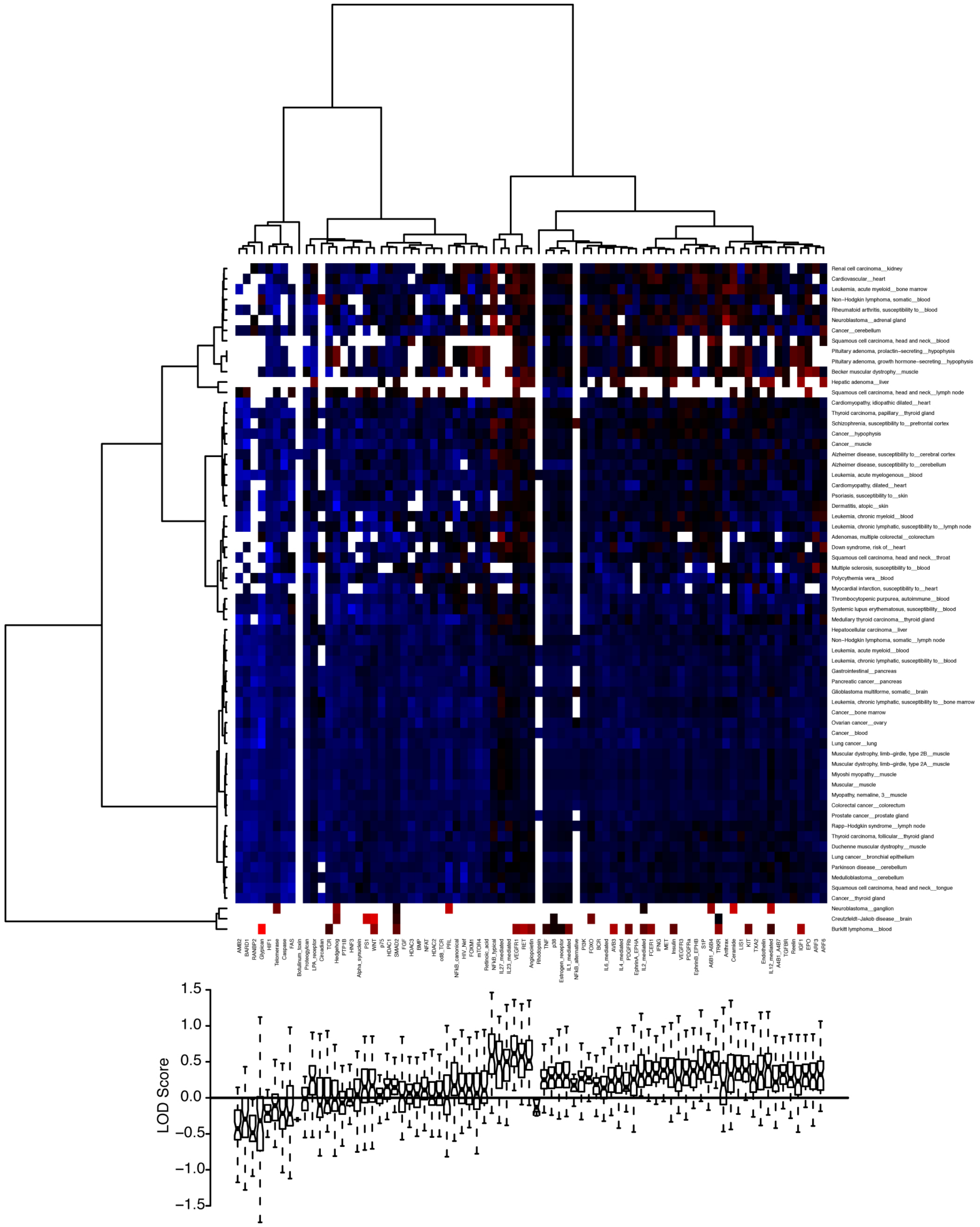

Supplementary Table 1: Core set of signaling pathways.

Pathway	Prediction tools
Rhodopsin	4/6
Botulinum	5/6
TGFBR	1/6
BMP	5/6
IGF1	3/6
VEGFR3	2/6
EphrinB/EPHB	2/6
PDGFa	4/6
MET	2/6
EphrinA/EPHA	3/6
RET	4/6
VEGFR1	2/6
REELIN	4/6
TRKR	1/6
mTOR4	2/6
EPO	1/6

Supplementary Table 1 Core set of signaling pathways. Prediction tools show the fraction of different tools having the corresponding pathway within the top cluster.

Supplementary Table 2: Core set of signaling pathways obtained by the cancer related microRNAs.

Pathway	Prediction tools
Rhodopsin	5/6
Botulinum	5/6
BMP	4/6
IGF1	3/6
VEGFR3	1/6
EphrinB/EPHB	2/6
PDGFa	3/6
MET	2/6
EphrinA/EPHA	3/6
RET	2/6
VEGFR1	1/6
REELIN	4/6
TRKR	1/6
mTOR4	1/6

Supplementary Table 2 Core set of signaling pathways obtained by the cancer related microRNAs. Prediction tools show the fraction of different tools having the corresponding pathway within the top cluster.

Supplementary Table 3: Core set of signaling pathways obtained by the non-cancer related microRNAs.

Pathway	Prediction tool
Rhodopsin	5/6
NFκB	5/6
VEGFR1	2/6
TGFBR	2/6
IGF1	2/6
EphrinB/EPHB	2/6
KIT	1/6
TRKR	2/6
PDGFa	5/6
VEGFR1	1/6
mTOR4	1/6
EPO	2/6
MET	3/6
RET	2/6

Supplementary Table 3 Core set of signaling pathways obtained by the non-cancer related microRNAs. Prediction tool shows the fraction of different tools having the corresponding pathway within the top cluster.

Cellular location analysis

Supplementary Figure 6: Comparison between different microRNA prediction tools

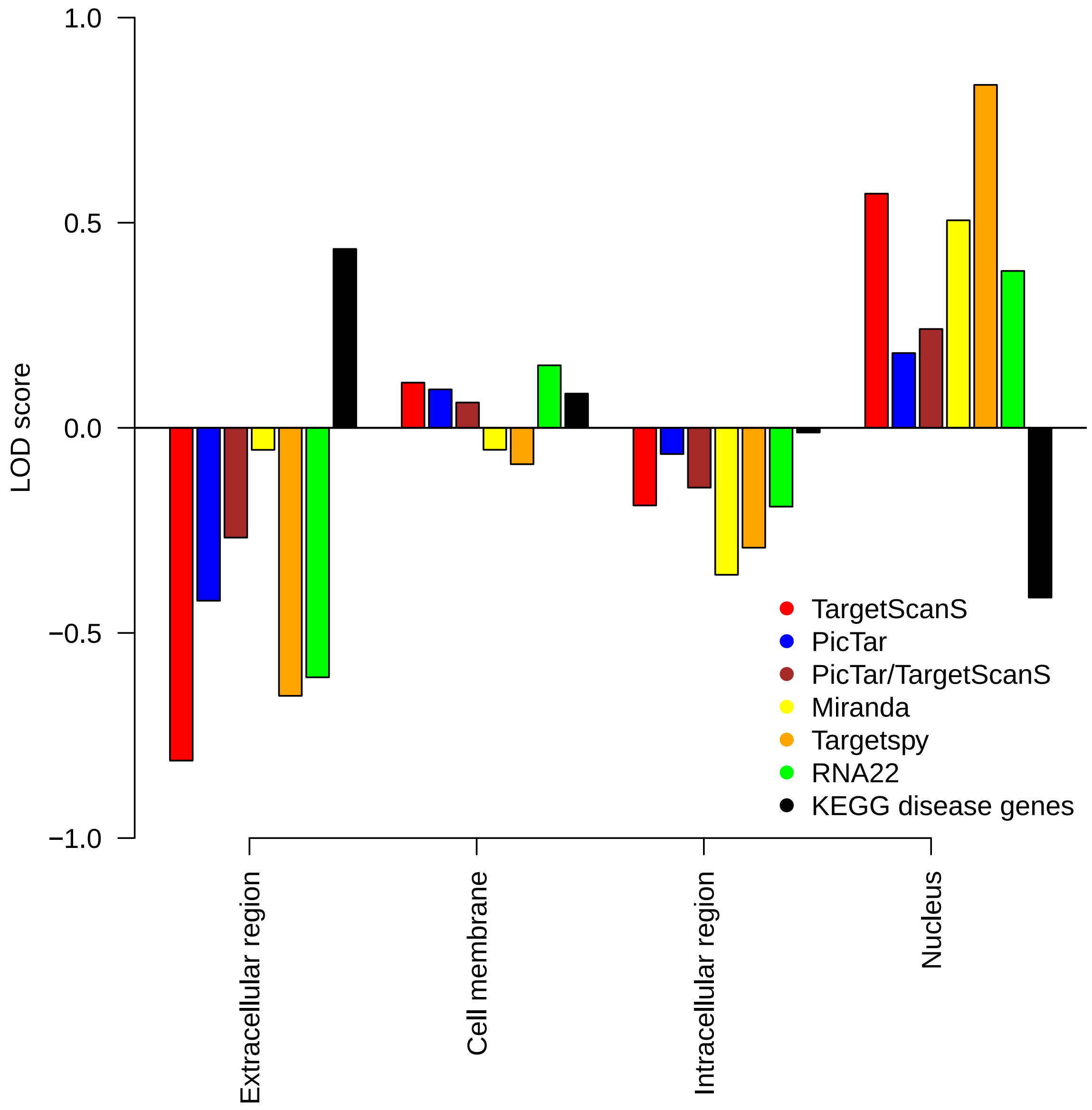

Supplementary Figure 6 Observed LOD scores for cellular location of several microRNA prediction methods (Intersection of PicTar and TargetScanS, TargetScanS, PicTar, Miranda, TargetSpy, and RNA22) and KEGG DISEASE proteins. Different features like conservation of the seed region (e.g. TargetScanS) as well as binding energies (e.g. Miranda) are taken into account to predict microRNA-transcript interactions. Based on differences in these prediction methods the overlap between the targets from different tools is low [2]. In this work, it was also shown that Miranda has similar high sensitivity compared to the top method like TargetScanS, but exhibit a substantial increase in the number of total predictions. This could be one explanation why Miranda shows a different result for microRNA targets in extracellular and intracellular regions compared to the remaining prediction tools, which show very similar results. The findings indicate robustness of our results, independent on the prediction tools. In addition, this findings support our result of complementary behavior of KEGG DISEASE proteins and microRNA targets.

Supplementary Figure 7: Comparison between different disease sets

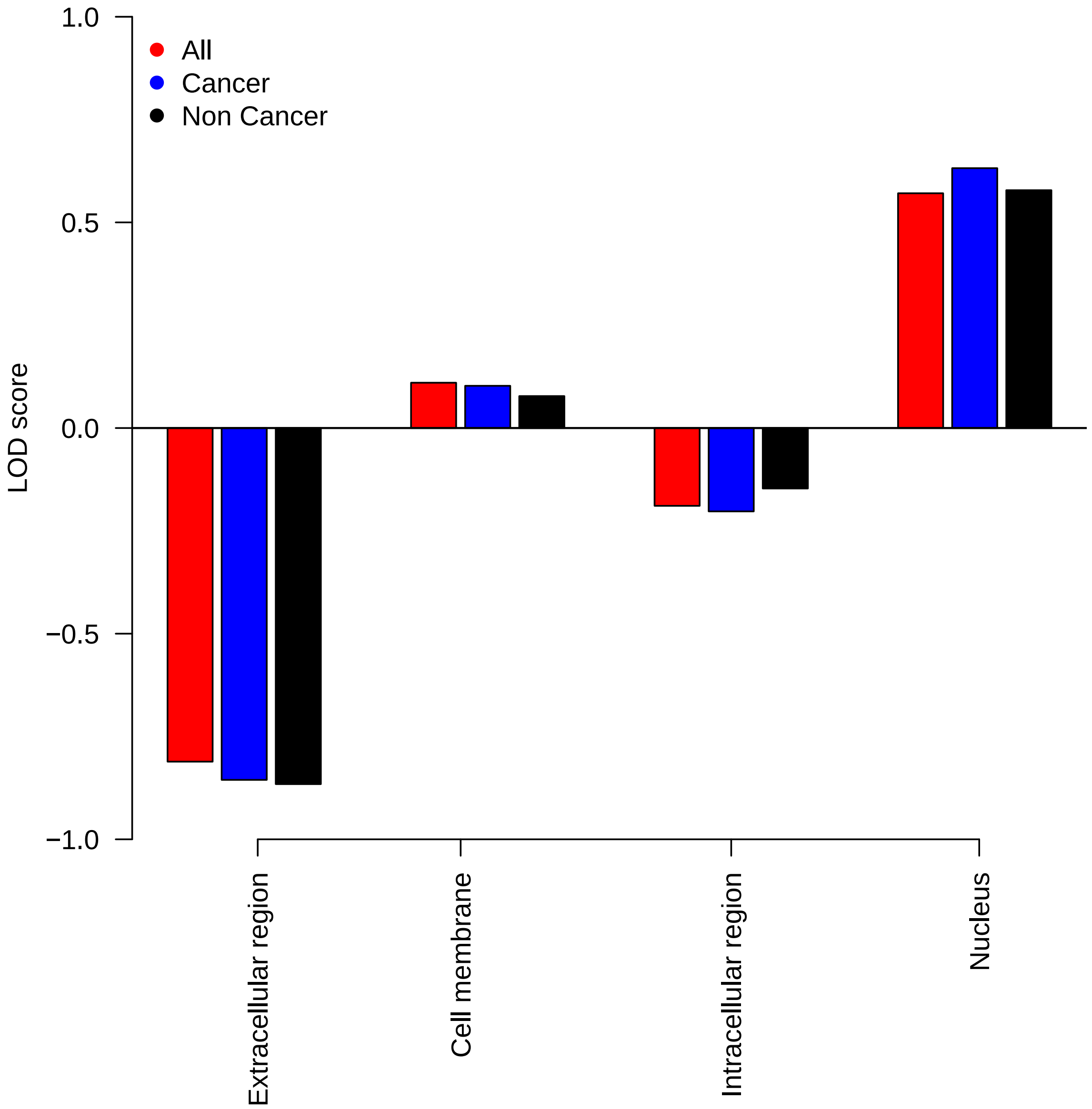

Supplementary Figure 7 Observed LOD scores for cellular location of all disease-associated microRNA targets and two subsets of diseases (Cancer, Non Cancer) using TargetScanS. For cancer and non cancer, we observed similar scores compared to scores obtained by using all diseases showing that the location pattern is rather a common result and not depended on the subsets of cancer and non-cancer related microRNAs.

Supplementary Figure 8: Comparison between different disease gene sets

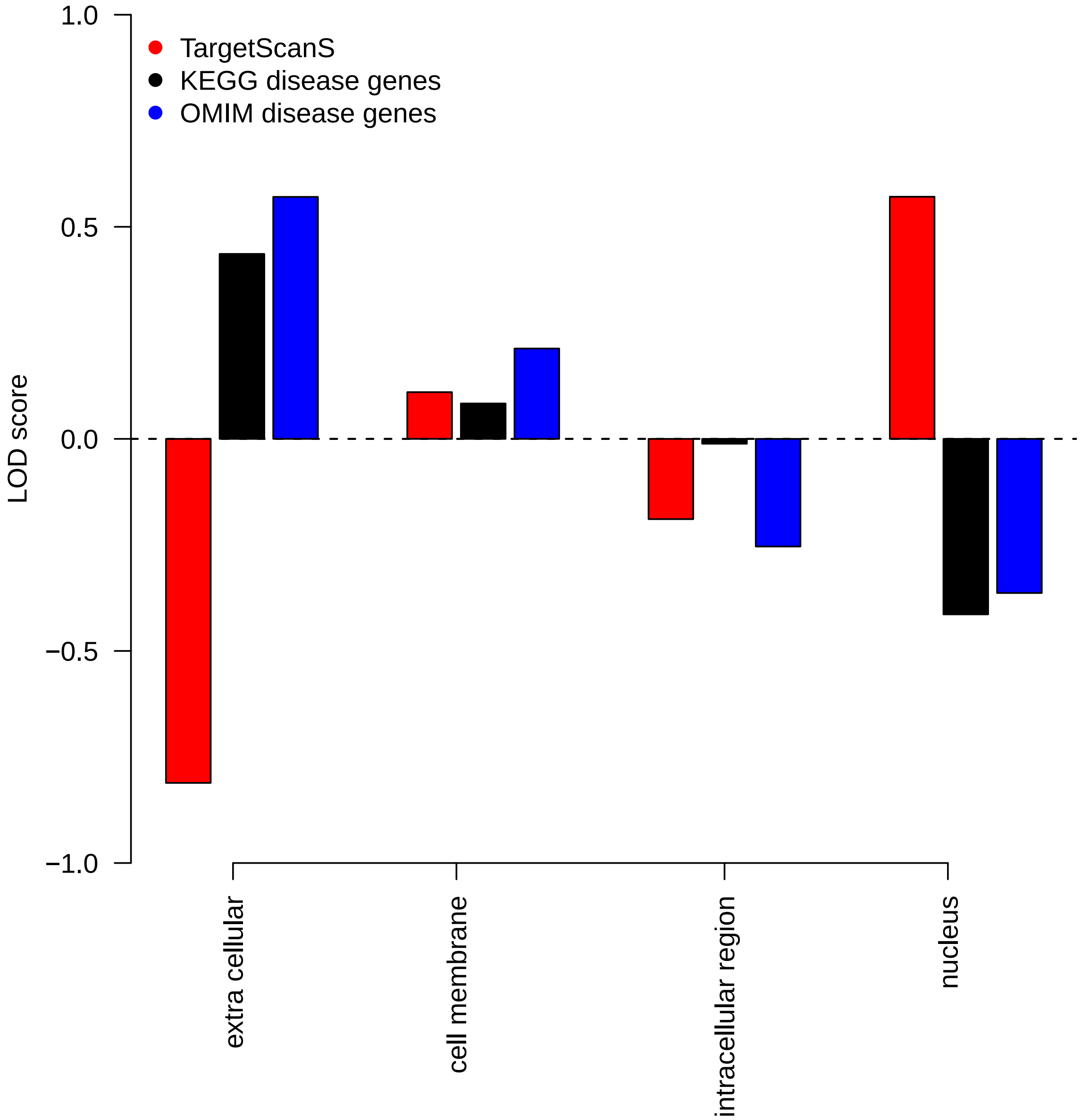

Supplementary Figure 8 Observed LOD scores for cellular location of microRNA targets and two sets of disease-associated genes (KEGG DISEASE and OMIM). For OMIM, we observed similar scores compared to KEGG DISEASE proteins that confirms our finding and shows robustness of our results. In addition, this finding supports our result of complementary behavior of disease-associated genes (KEGG DISEASE and OMIM) and microRNA targets.

Process type analysis

Supplementary Figure 9: Process type analysis using different microRNA prediction tools

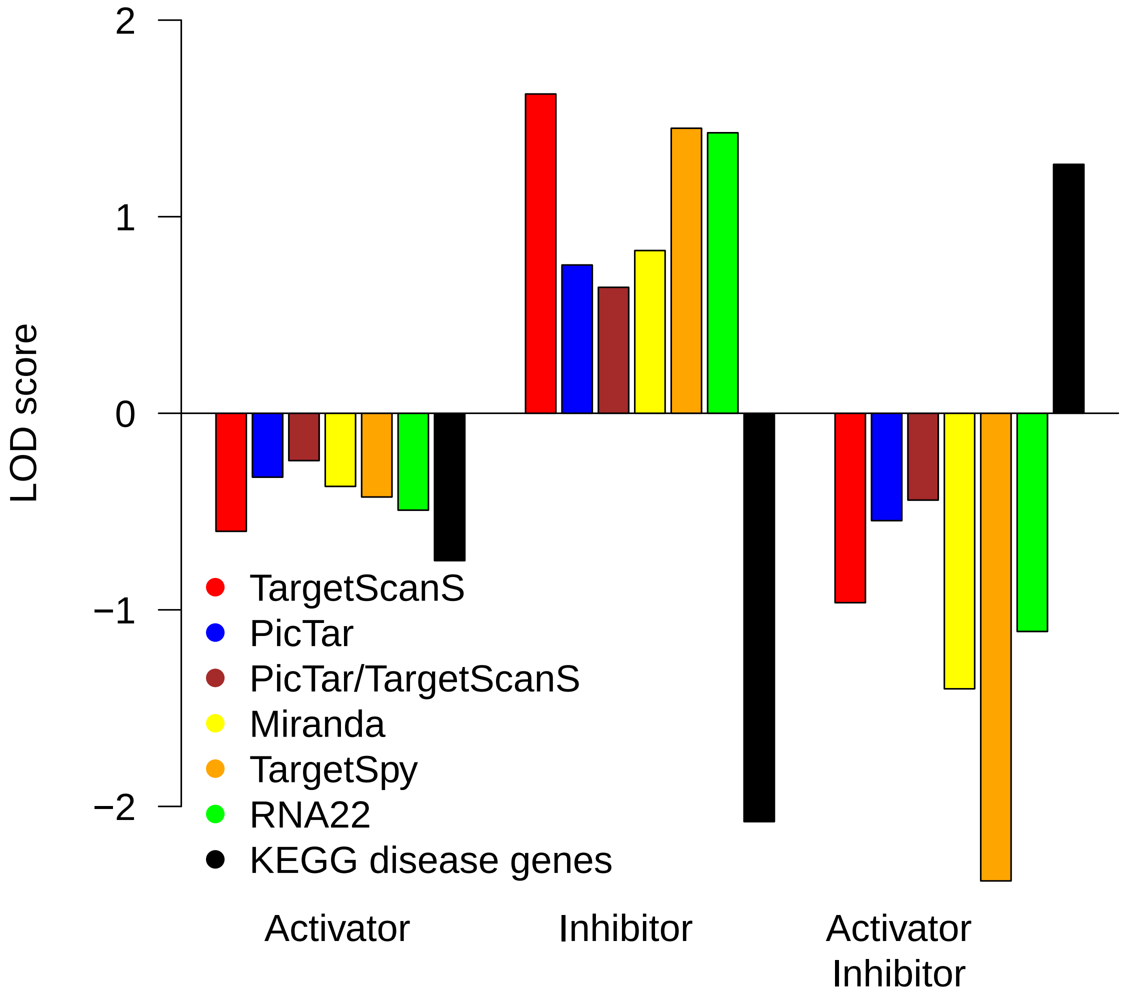

Supplementary Figure 9 Observed LOD scores for process type behavior of several microRNA prediction methods (Intersection of PicTar and TargetScanS, TargetScanS, PicTar, Miranda, TargetSpy, and RNA22) and KEGG DISEASE proteins. We obtained similar results for the enrichment of microRNA targets acting as inhibitors. These results confirm our finding of an inhibitory effect of microRNA-targets. In addition, this finding supports our result of complementary behavior of KEGG DISEASE proteins and microRNA targets.

Supplementary Figure 10: Comparison between different disease sets

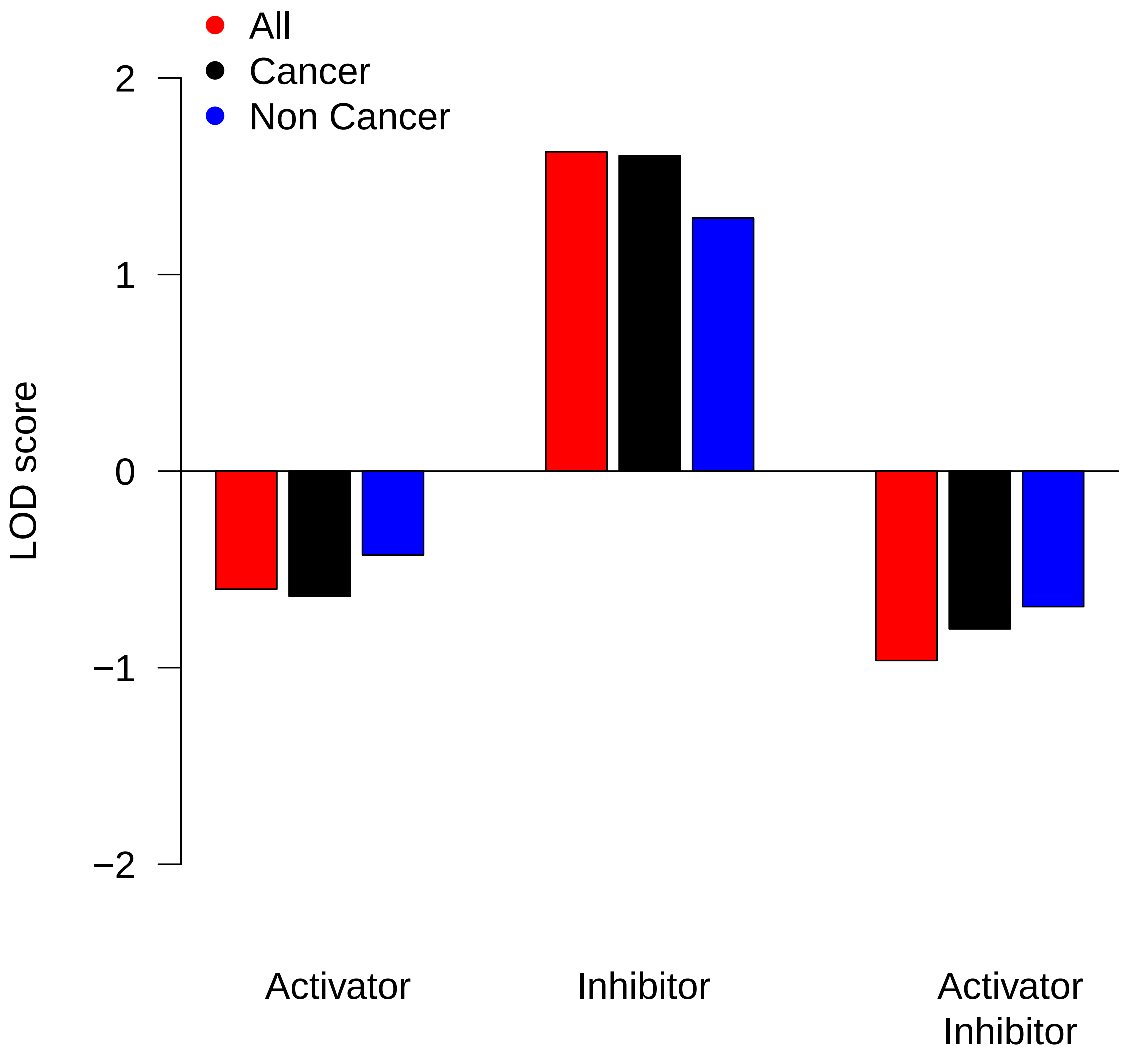

Supplementary Figure 10 Observed LOD scores for process type behavior of all disease-associated microRNA targets and two subsets of diseases (Cancer, Non Cancer) using TargetScanS. For cancer and non cancer, we observed similar scores compared to scores obtained by using all diseases showing that the process type behavior is rather a common result and not depended on the subsets of cancer and non-cancer related microRNAs.

Supplementary Figure 11: Comparison between different disease gene sets

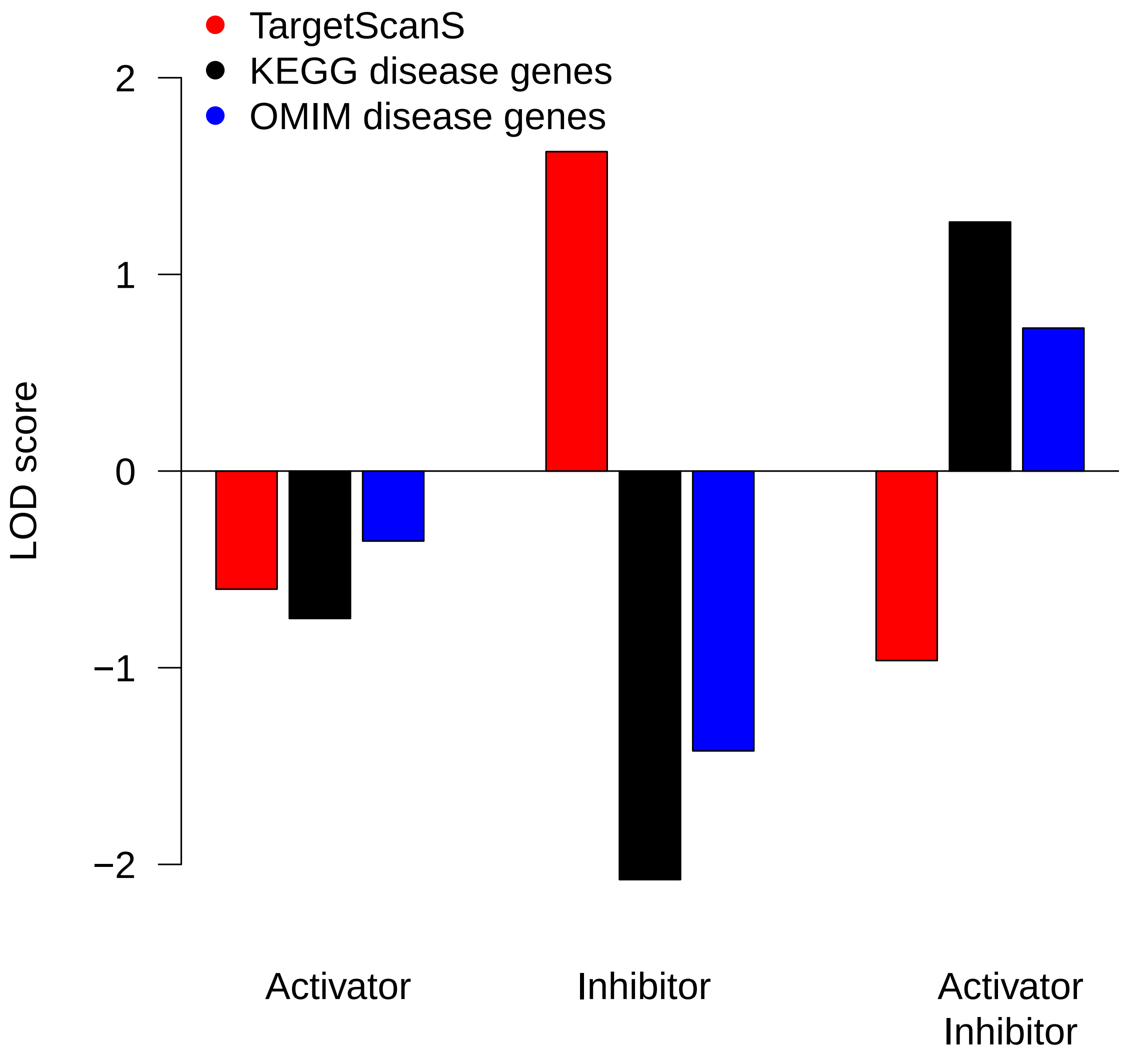

Supplementary Figure 11 Observed LOD scores for process type behavior of microRNA targets and two sets of disease-associated genes (KEGG DISEASE and OMIM). For OMIM, we observed similar scores compared to KEGG DISEASE proteins that confirms our finding. In addition, this finding supports our result of complementary behavior of disease-associated genes (KEGG DISEASE and OMIM) and microRNA targets.

References

[1] Hausser J, Landthaler M, Jaskiewicz L, Gaidatzis D, Zavolan M: **Relative contribution of sequence and structure features to the mRNA binding of Argonaute/EIF2C-miRNA complexes and the degradation of miRNA targets.** *Genome research* 2009, **19**(11):2009–20, [http://www.ncbi.nlm.nih.gov/pubmed/19767416].

[2] Sethupathy P, Megraw M, Hatzigeorgiou AG: **A guide through present computational approaches for the identification of mammalian microRNA targets.** *Nat Methods* 2006, **3**:881–886, [http://dx.doi.org/10.1038/nmeth954].

[3] Ritchie W, Flamant S, Rasko JEJ: **Predicting microRNA targets and functions: traps for the unwary.** *Nat Methods* 2009, **6**:397–398.
