# Supplementary figures and images for: Tissue-Specific Target Analysis of Disease-Associated MicroRNAs in Human Signaling Pathways
Source: PLoS One. 2010 Jun 30;5(6):e11154. doi: 10.1371/journal.pone.0011154 (PMC2894853; doi:10.1371/journal.pone.0011154)

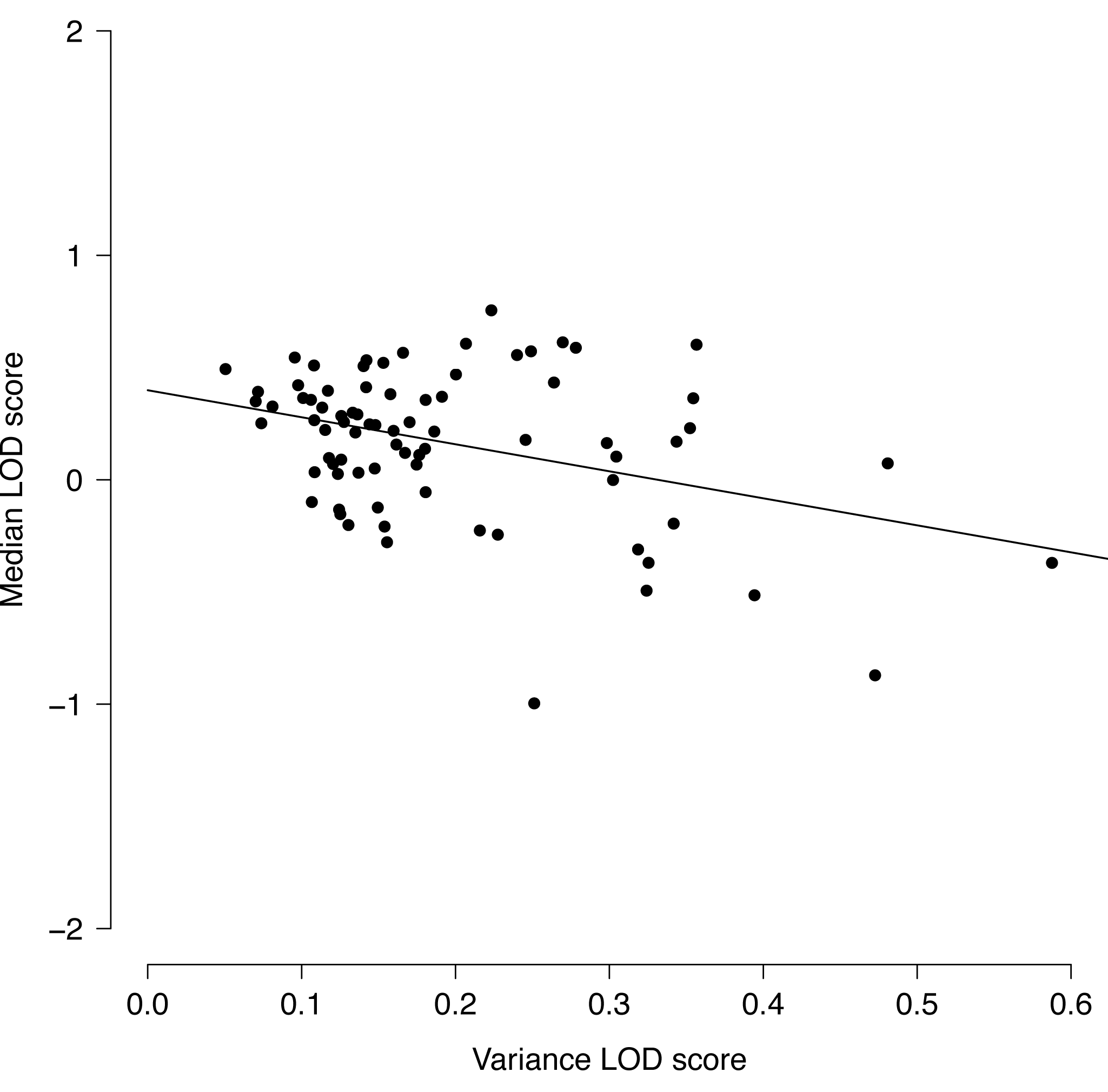

Supplement: Figure S1 — Anticorrelation of median LOD score against variance for signaling pathways. We obtained a significant negative correlation (Pearson correlation coefficient Cp = −0.37, P = 0.007). The result implies that deregulated microRNAs in human diseases target the same set of signaling pathways irrespective of the specific disorder. The results of the linear regressions is shown by the black line. (0.12 MB TIF) [file pone.0011154.s002.tif]

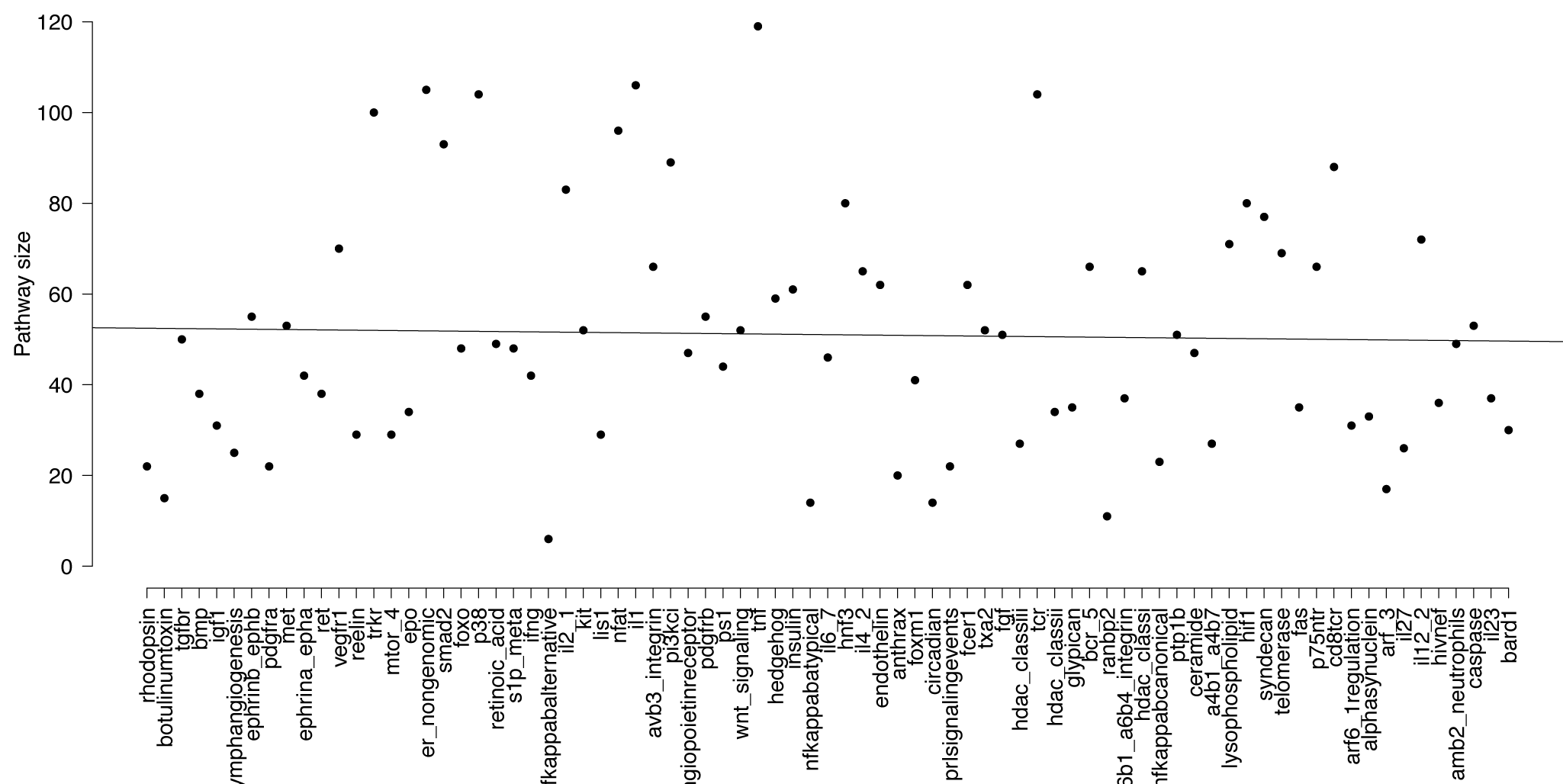

Supplement: Figure S2 — Pathway size against pathways ordered by median LOD score. We found no correlation (Cp = −0.032, P = 0.83) between pathway size against pathways ordered by median LOD score. The results of the linear regressions is shown by the black line. (0.15 MB TIF) [file pone.0011154.s003.tif]

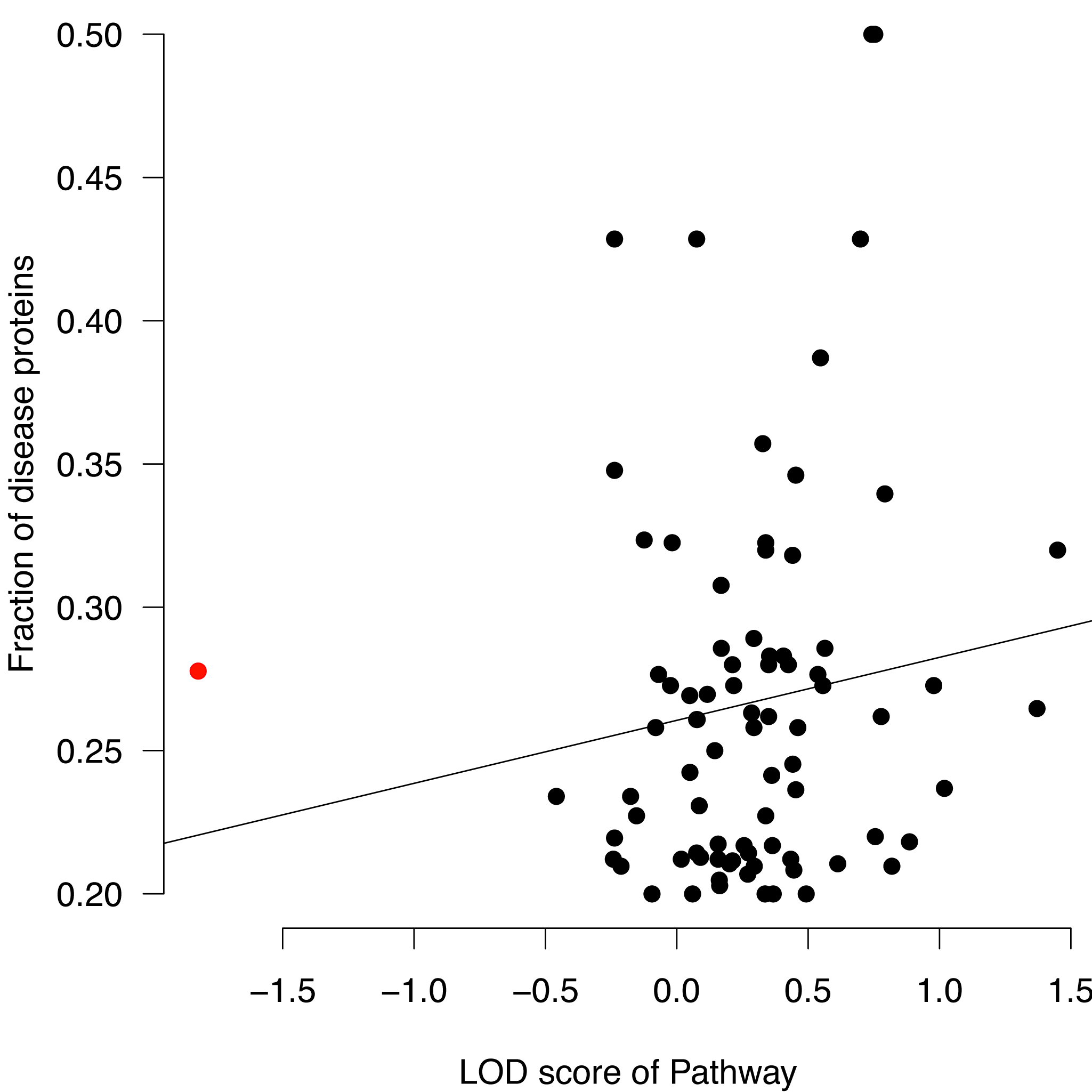

Supplement: Figure S3 — Correlation between KEGG DISEASE proteins and microRNAs in signaling pathways, using 24 diseases both annotated in PhenomiR and KEGG DISEASE. Median LOD score of signaling pathways against the fraction of disease-associated pathway proteins (Cp = 0.14, P = 0.21). We consider all pathways showing a fraction of disease-associated proteins ≥0.2. We observe no significant correlation between increasing LOD scores and the fraction of disease proteins even if we exclude the outlier (marked in red) (Cp = 0.18, P = 0.127). The results of the linear regressions is shown by the black line. (0.15 MB TIF) [file pone.0011154.s004.tif]

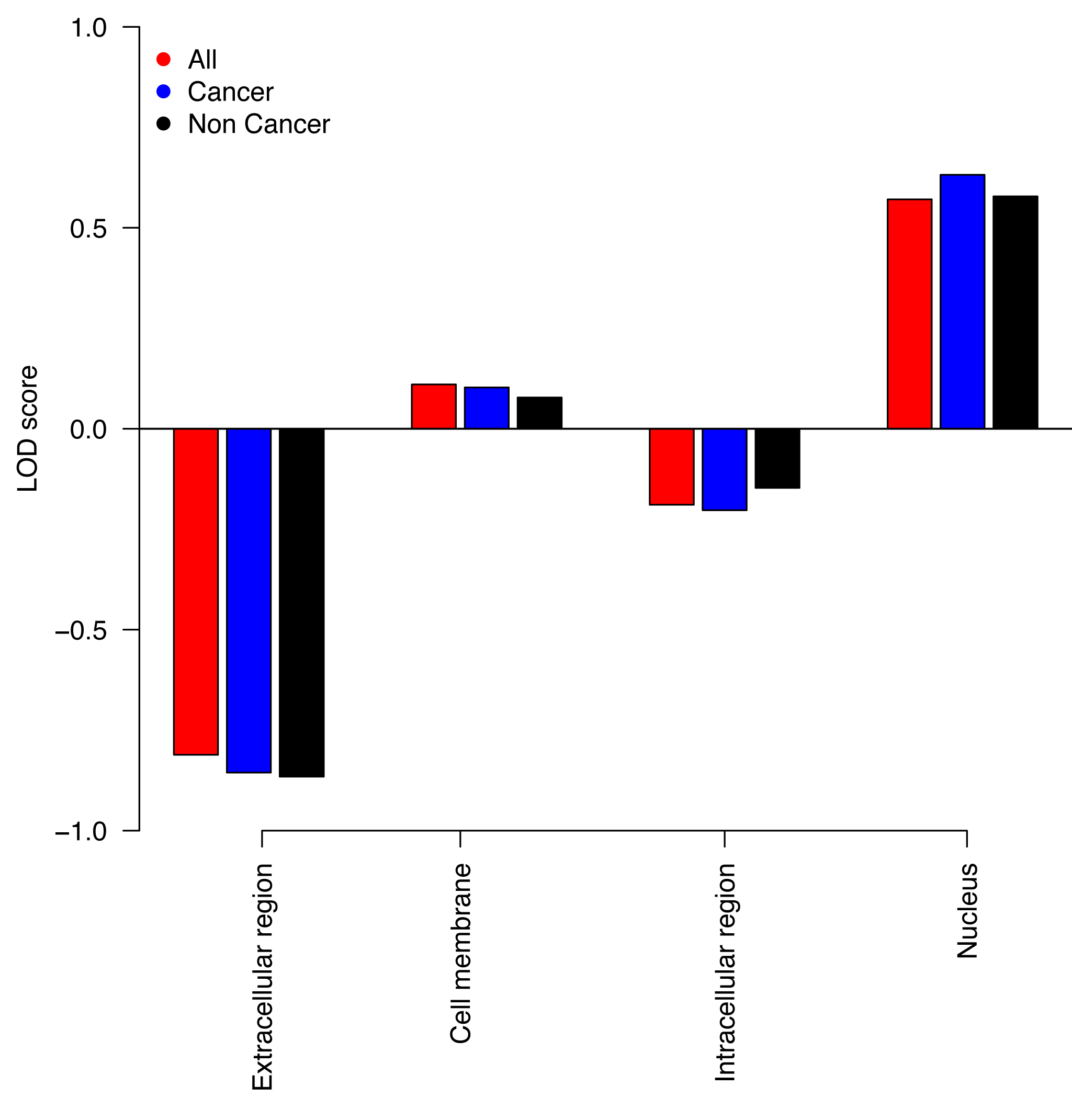

Supplement: Figure S4 — Comparison between different disease sets. Observed LOD scores for cellular location of all disease-associated microRNA targets and two subsets of diseases (Cancer, Non Cancer) using TargetScanS. For cancer and non cancer, we observed similar scores compared to scores obtained by using all diseases showing that the location pattern is rather a common result and not depended on the subsets of cancer and non-cancer related microRNAs. (0.14 MB TIF) [file pone.0011154.s005.tif]

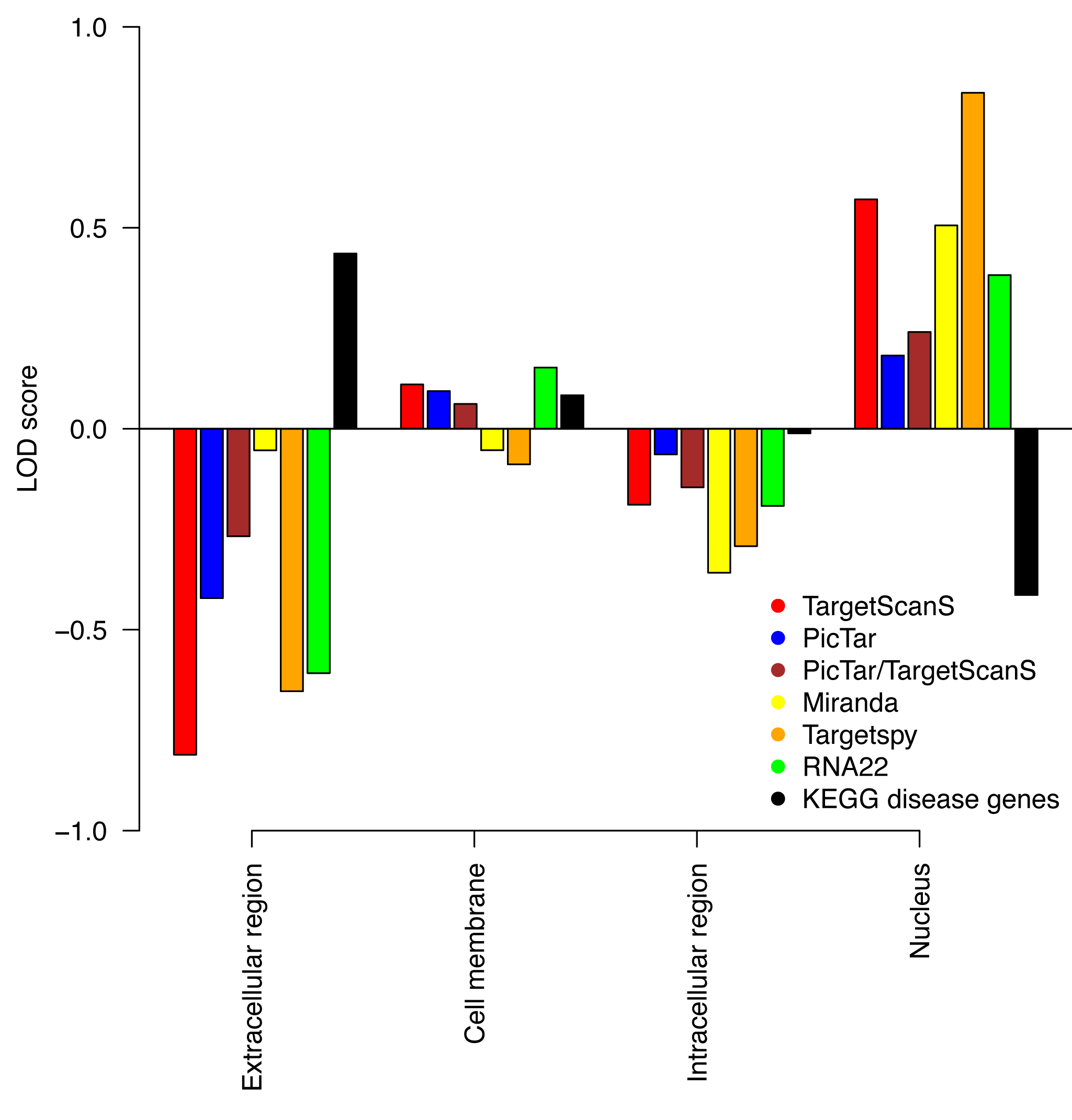

Supplement: Figure S5 — Comparison between different microRNA prediction tools. Observed LOD scores for cellular location of several microRNA prediction methods (Intersection of PicTar and TargetScanS, TargetScanS, PicTar, Miranda, TargetSpy, and RNA22) and KEGG DISEASE proteins. Different features like conservation of the seed region (e.g., TargetScanS) as well as binding energies (e.g., Miranda) are taken into account to predict microRNA-transcript interactions. Based on differences in these prediction methods the overlap between the targets from different tools is low (Sethupathy, 2006). In this work, it was also shown that Miranda has similar high sensitivity compared to the top method like TargetScanS, but exhibit a substantial increase in the number of total predictions. This could be one explanation why Miranda shows a different result for microRNA targets in extracellular and intracellular regions compared to the remaining prediction tools, which show very similar results. The findings indicate robustness of our results, independent on the prediction tools. In addition, this findings support our result of complementary behavior of KEGG DISEASE proteins and microRNA targets. (0.19 MB TIF) [file pone.0011154.s006.tif]

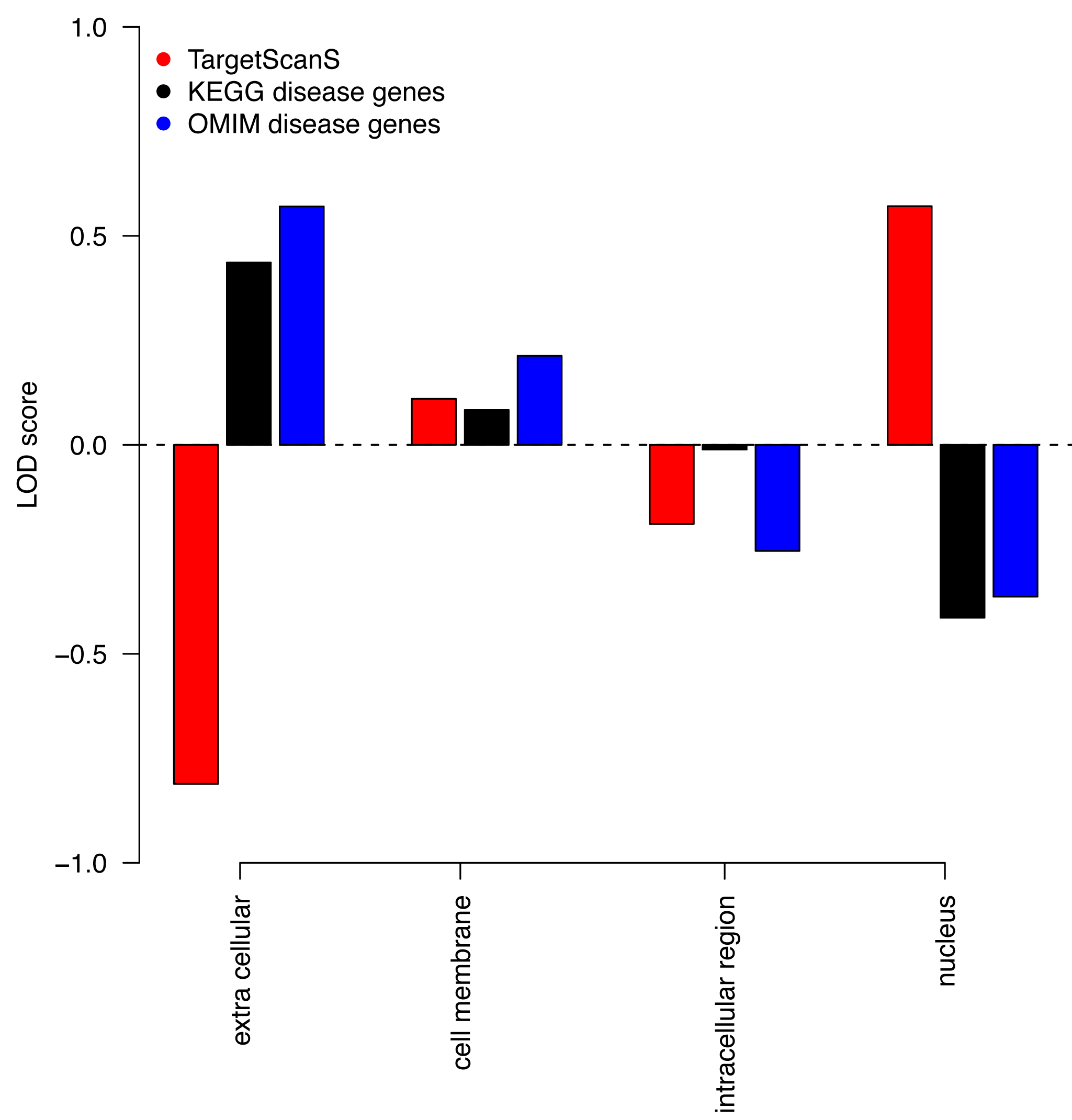

Supplement: Figure S6 — Comparison between different disease gene sets. Observed LOD scores for cellular location of microRNA targets and two sets of disease-associated genes (KEGG DISEASE and OMIM). For OMIM, we observed similar scores compared to KEGG DISEASE proteins that confirms our finding and shows robustness of our results. In addition, this finding supports our result of complementary behavior of disease-associated genes (KEGG DISEASE and OMIM) and microRNA targets. (0.15 MB TIF) [file pone.0011154.s007.tif]

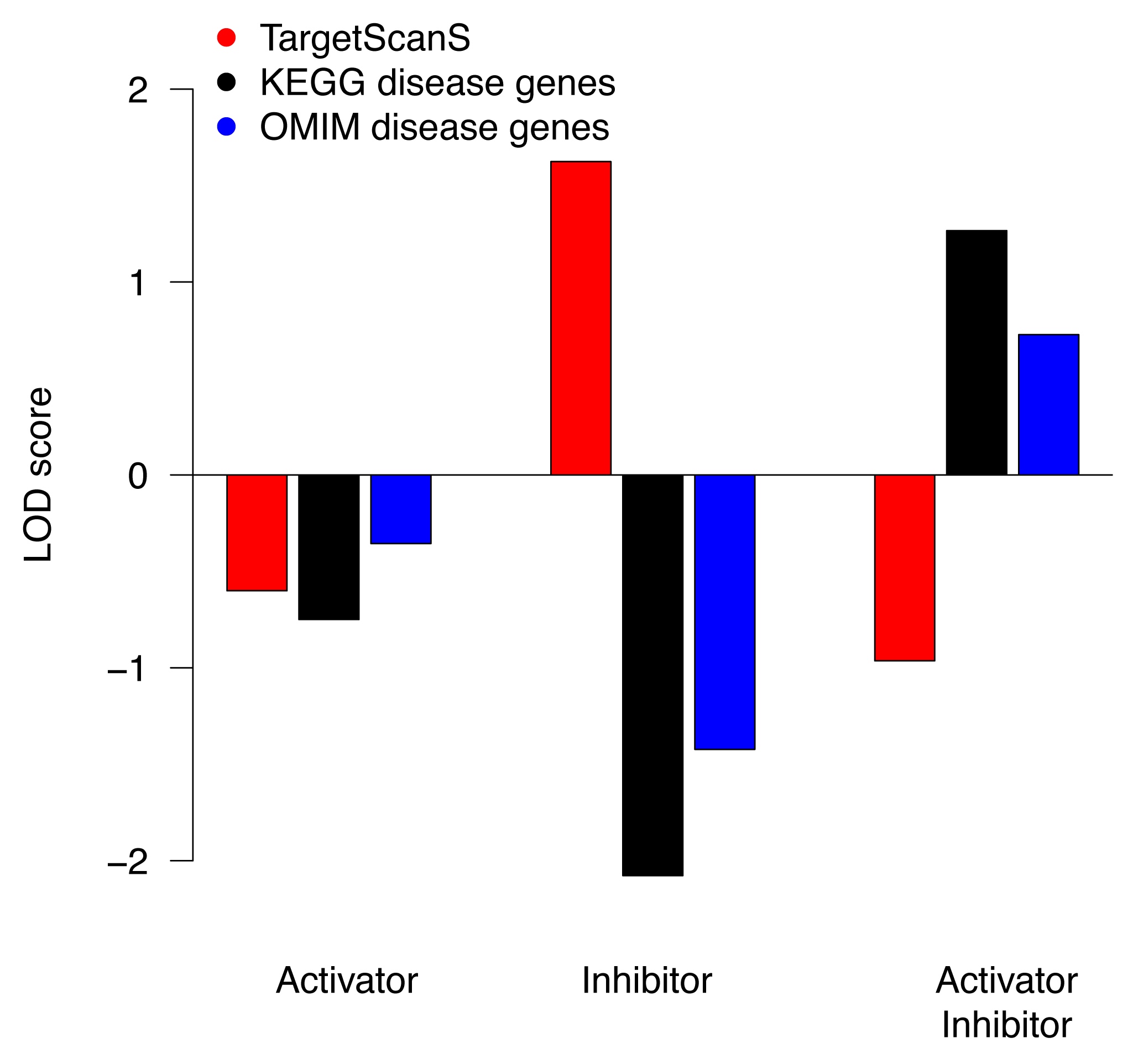

Supplement: Figure S7 — Comparison between different disease gene sets. Observed LOD scores for process type behavior of microRNA targets and two sets of disease-associated genes (KEGG DISEASE and OMIM). For OMIM, we observed similar scores compared to KEGG DISEASE proteins that confirms our finding. In addition, this finding supports our result of complementary behavior of disease-associated genes (KEGG DISEASE and OMIM) and microRNA targets. (0.15 MB TIF) [file pone.0011154.s008.tif]
